# A positive-strand RNA virus uses alternative protein-protein interactions within a viral protease/cofactor complex to switch between RNA replication and virion morphogenesis

**DOI:** 10.1371/journal.ppat.1006134

**Published:** 2017-02-02

**Authors:** Danilo Dubrau, M. Alejandra Tortorici, Félix A. Rey, Norbert Tautz

**Affiliations:** 1 Institute of Virology and Cell Biology, University of Luebeck, Luebeck, Germany; 2 Institut Pasteur, Unité de Virologie Structurale, Paris, France; 3 CNRS UMR 3569 Virologie, Paris, France; University of Pennsylvania School of Medicine, UNITED STATES

## Abstract

The viruses of the family *Flaviviridae* possess a positive-strand RNA genome and express a single polyprotein which is processed into functional proteins. Initially, the nonstructural (NS) proteins, which are not part of the virions, form complexes capable of genome replication. Later on, the NS proteins also play a critical role in virion formation. The molecular basis to understand how the same proteins form different complexes required in both processes is so far unknown. For pestiviruses, uncleaved NS2-3 is essential for virion morphogenesis while NS3 is required for RNA replication but is not functional in viral assembly. Recently, we identified two gain of function mutations, located in the C-terminal region of NS2 and in the serine protease domain of NS3 (NS3 residue 132), which allow NS2 and NS3 to substitute for uncleaved NS2-3 in particle assembly. We report here the crystal structure of pestivirus NS3-4A showing that the NS3 residue 132 maps to a surface patch interacting with the C-terminal region of NS4A (NS4A-kink region) suggesting a critical role of this contact in virion morphogenesis. We show that destabilization of this interaction, either by alanine exchanges at this NS3/4A-kink interface, led to a gain of function of the NS3/4A complex in particle formation. In contrast, RNA replication and thus replicase assembly requires a stable association between NS3 and the NS4A-kink region. Thus, we propose that two variants of NS3/4A complexes exist in pestivirus infected cells each representing a basic building block required for either RNA replication or virion morphogenesis. This could be further corroborated by *trans*-complementation studies with a replication-defective NS3/4A double mutant that was still functional in viral assembly. Our observations illustrate the presence of alternative overlapping surfaces providing different contacts between the same proteins, allowing the switch from RNA replication to virion formation.

## Author summary

Many positive-strand RNA viruses replicate without transcribing subgenomic RNAs otherwise often used to temporally coordinate the expression of proteins involved either in genome replication (early) or virion formation (late). Instead, the RNA genomes of the *Flaviviridae* are translated into a single polyprotein. Their nonstructural proteins (NS), while not present in the virions, are known to be crucially involved in RNA replication and virion formation. The important question how the same proteins form specific complexes required for fundamentally different aspects of the viral replication cycle is not solved yet. For pestiviruses the mature NS3/4A complex is an essential component of the viral RNA-replicase but is incapable of participating in virion morphogenesis which in turn depends on uncleaved NS2-3 in complex with NS4A. However, a gain of function mutation in NS3 enabled the NS3/4A complex to function in virion assembly. Using structure guided mutagenesis in combination with functional studies we identified the interface between NS3 and the C-terminal NS4A region as a module critical for the decision whether a NS3/4A complex serves in RNA replication or as a packaging component. Thus, we propose that subtle changes in local protein interactions represent decisive switches in viral complex formation pathways.

## Introduction

The *Flaviviridae* family comprises positive-strand RNA viruses and consists of four genera, *Flavivirus*, *Pegivirus*, *Hepacivirus*, and *Pestivirus*, with the latter three genera showing a significantly higher degree of similarity [1–3]. Pestiviruses, like bovine viral diarrhea virus (BVDV-1 and -2) and classical swine fever virus (CSFV) are important animal pathogens which cause significant economic damage in livestock industries [3].

The RNA genomes of the *Flaviviridae* encompass one single open reading frame (ORF), which is flanked by the 5’ and 3’ untranslated regions (UTR) [4]. Upon infection of the host cell the viral RNA genome is translated into a polyprotein that is processed by cellular and viral proteases into the mature structural (SP) and nonstructural (NS) proteins. For members of the genus *Pestivirus* the array in the polyprotein is the following: NH_2_-N^pro^ (N-terminal autoprotease), C (capsid protein, core), E^rns^ (envelope protein RNase secreted), E1, E2, p7, NS2-3 (NS2 and NS3), NS4A, NS4B, NS5A, NS5B-COOH [4]. The N-terminal autoprotease N^pro^ generates its own C terminus and thereby the N terminus of the capsid protein core (C). Further cleavages releasing the structural proteins C, E^rns^, E1 and E2 as well as p7 are mediated by proteases residing in the endoplasmatic reticulum (ER) [4, 5].

The cleavage between NS2 and NS3 is catalyzed by an autoprotease in NS2 [6]. The activity of the NS2 protease is temporally regulated by a cellular cofactor leading to significant amounts of uncleaved NS2-3 in pestivirus infected cells (see below).

The cleavages downstream of NS3, NS4A, NS4B and NS5A are catalyzed by the serine protease domain of NS3 which requires NS4A as cofactor for full proteolytic activity and is termed NS3-4A protease [7–10]. Co-immunoprecipitation experiments have shown that NS4A forms a stable complex with NS3 (NS3/4A complex). The N-terminal part of NS4A is highly hydrophobic and in analogy to HCV NS4A most likely comprises a transmembrane domain. The most C-terminal region is not required for the protease cofactor function of NS4A [7]. For HCV NS4A it was observed that the central part of NS4A stably intercalates into the N-terminal beta-barrel domain of the NS3 serine protease domain, a process required to gain full NS3 protease activity [11, 12]. The downstream part of HCV NS4A is subdivided into the kink region followed by the acidic domain [13, 14]. This nomenclature will be also used for the pestiviral NS4A. In addition to its protease function, NS3 of HCV and pestiviruses comprises helicase and NTPase activity [15–17]. Only when NS2 is cleaved off, NS3 is capable of forming the functional viral RNA-replicase together with the viral proteins NS4A, NS4B, NS5A, NS5B and an unknown number of cellular factors [4, 6, 18–20].

For the members of the *Flaviviridae* the processes involved in virion morphogenesis are not completely understood, it is however known that an interplay between the structural and nonstructural proteins is crucial [21]. For HCV all NS proteins are involved in this process with NS2 being of special importance. It interacts with both structural and nonstructural proteins and thus is proposed to represent a platform for virion assembly [22–27].

For pestiviruses, all NS proteins have been shown to be involved in virion formation, with the exception of N^pro^ and NS4B [19, 28–31]. Since NS4B is critically involved in virion morphogenesis of HCV [32, 33], it seems likely that its pestiviral counterpart is also required for this process.

A special feature of pestiviruses is the existence of significant amounts of uncleaved NS2-3 in the infected cell and its essential role in virion formation. A peculiarity of the pestiviral life cycle is the temporal restriction of NS2-3 processing by the NS2 autoprotease [34, 35]. This regulation is based on the fact that the activity of the pestiviral NS2 protease depends on the cellular cofactor Jiv (J-domain protein interacting with viral protein, also termed DNAJC14) [6, 29, 34, 36]. Due to the limited amounts of Jiv in the infected host cell, NS2-3 cleavage is mostly restricted to the early phase of infection. NS2-3 translated at later time points is only inefficiently processed leading to the accumulation of uncleaved NS2-3 which temporally correlates with the onset of virion morphogenesis. A second important aspect connected to the downregulation of NS2-3 processing is its crucial role for the non-cytopathogenic (ncp) biotype of pestiviruses in cell culture. Upregulation of the levels of free NS3, e.g. by the insertion of an ubiquitin (Ubi) moiety between NS2 and NS3, results in a cp biotype and interferes with the capability of BVDV to establish persistent infections in cattle [3, 37–40].

With regard to virion morphogenesis, previous studies on BVDV and CSFV have shown that the introduction of an internal ribosomal entry site (IRES) or an ubiqutin coding sequence between the sequences coding for NS2 and NS3 result in a complete loss of infectious particle formation [28, 29]. Based on these and other studies [6] it became clear that NS3/4A or uncleaved NS2-3 in complex with NS4A (NS2-3/4A) represent essential components of complexes which facilitate either RNA replication or virion formation: While NS3/4A is an essential part of the RNA replication complex, only NS2-3/4A can facilitate virus assembly.

The essential role of NS2-3/4A in the pestiviral life cycle is remarkable when compared to the closely related HCV. In cell culture, the cleavage of HCV NS2-NS3 is highly efficient and previous studies demonstrated that HCV genomes that encode an IRES insertion between NS2 and NS3 were not severely affected with regard to infectious particle formation [26, 41].

This finding inspired earlier work by Lattwein, Klemens and coworkers [42, 43] who succeeded in adapting a pestivirus to virion morphogenesis in absence of uncleaved NS2-3, similar to the situation described for HCV. Surprisingly, this adaptation of a variant of BVDV-1 strain NCP7 that encodes an ubiquitin monomer between NS2 and NS3 (NS2-Ubi-NS3) required just two amino acid exchanges to allow for efficient particle formation in the absence of uncleaved NS2-3 [43]. One amino acid exchange was located in the C-terminal part of the NS2 protease (2/E440V –originally termed E1576V due to its position in the polyprotein), whereas the other exchange was found in the NS3 protease domain (3/V132A –originally termed as V1721A) [43].

In the present study, we addressed the molecular basis of the gain of function observed for NS2 and the NS3/4A complex in virion morphogenesis. In the absence of structural data for NS2 we focused on the mutation of residue 132 of the NS3 protease domain. We report here the crystal structure of a single chain construct encompassing full-length pestivirus NS3 in complex with NS4A residues 21–57. The data revealed that the gain of function mutation in NS3 maps to a hydrophobic surface patch which interacts with the NS4A-kink region. Our structure-guided studies suggest that NS3/4A can adopt two different conformations in the infected cell–a closed form that is used in RNA replication complexes and a more open conformation functional in viral assembly. Furthermore, our data indicate that the NS2-3/4A complex, required for virion assembly of prototype pestiviruses, displays a similar open conformation.

Taken together, our study revealed a novel mechanism by which protein complexes adopt alternative conformations to serve in fundamentally different processes such as RNA replication and virion morphogenesis. We believe that our data will stimulate future analyses of other positive-strand RNA viruses, since it seems likely that similar mechanisms will be used to endow viral protein complexes with the capacity to function in genome replication and virion morphogenesis, a task common to so many positive-strand RNA viruses.

## Results

### Expression, purification and biochemical characterization of the CSFV NS3/4A complex

**Fig 1:**
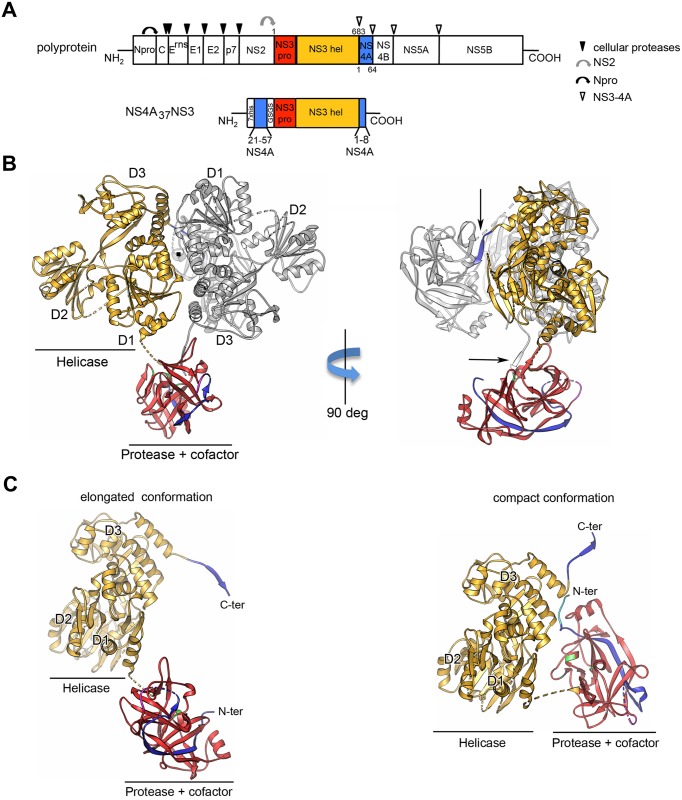
Crystal structure of the CSFV NS3/4A complex. (A) Schematic diagram of the pestivirus polyprotein indicating the individual mature proteins (boxes) and proteolytic cleavage sites (arrows) (top). Scheme of the construct NS4A_37_NS3 used for crystallization (bottom). (B) Ribbon diagram of the two molecules of CSFV NS4A_37_NS3 in the asymmetric unit in two orientations. The two molecules within the AU of the crystals are related by a local 2-fold axis (perpendicular to the plane of the figure) around which D1 and D3 of the two helicase domains make a head-to-tail crystallographic dimer interaction. The intersection of the 2-fold axis with the paper is represented here by a central black dot (left panel). Right panel shows an orthogonal view of the left panel. One molecule is colored following the color code use in panel (A): yellow: helicase domain, red: protease domain, blue: cofactor domain, pink: GSGS linker and the other one is colored in grey. The arrows in the right panel indicate that the C-terminal end of one protomer lies in the protease active site of the other. (C) Ribbon diagram of the two molecules present in the asymmetric unit showing the protease/cofactor complex (red/blue) relative to the helicase domain (yellow). The protease domain adopts two different orientations relative to the helicase domain, giving rise to an elongated and compact conformation of the enzyme. Catalytic residues in the protease domain are depicted in green.

To express soluble CSFV NS4A_37_NS3 for crystallization, we used recombinant constructs encoding full-length NS3 (residues 1 to 683) followed by the 8 N-terminal residues of NS4A; the NS3 sequence is preceeded by residues 21 to 57 of the protease cofactor NS4A (Fig 1A). These constructs corresponded to wild-type and single or double mutants, i.e. NS4A_37_NS3 carrying a mutation in the protease active site (S163A) or in addition, a mutation in the helicase active site (K232A). Wild-type and mutant proteins, previously characterized for helicase activity, express very well in *E*. *coli* and were purified as described earlier [44]. By an *in vivo trans*-cleavage assay described in S1 Fig we confirmed that wild-type NS4A_37_NS3 but not its derivative containing the active site mutation S163A has proteolytic activity (S1 Fig).

### Crystallization and structure determination

We determined the structure of the NS4A_37_NS3 active site mutant S163A as described in Materials and Methods and refined it to 3.05 Å resolution (PDB accession code: 5LKL). Table 1 lists the relevant crystallographic statistics, and S1 Table shows the disordered segments.

**Table 1:** Crystallographic statistics for CSFV NS4A_37_NS3 crystal.

**PDB accession code**	5LKL
**Crystallization conditions**	12% PEG 3.35 K, 250 mM tri-sodium citrate, 100 mM 2-aminoethanesulfonic acid
**Data Collection**	
Space group	P2_1_2_1_2
Unit cell a, b, c (Å) α = β = γ (°)	127.21, 168.84, 98.5, 90
Resolution range (Å)	38.95–3.05 (3.21–3.05)
Measured reflections	231905 (15766)
Unique reflections	40242 (5483)
Completeness (%)	98.3 (93)
Multiplicity (last shell)	5.8 (2.9)
(I) / σ(I) (last shell)	15.2 (1.8)
Rmerge (%) (last shell)*	9.1 (48.2)
**Refinement statistics**	
N° of reflections in refinement	40187
Molecules per asymmetric unit	2
R work, %^a^	19.74
R free, %^b^	23.65
N° of protein atoms	10789
N° of water molecules	10
Rms deviation from ideal values	
Bond lengths (Å)	0.005
Bond angles (°)	0.7
Overall average B factor, Å^2^ Ramachandran plot (%)^c^	83.4
Favored regions	97.35
Allowed regions	2.36
Outliers regions	0.29

^*a*^*R*merge = ∑(∑|*I*_i_ − 〈I〉| /∑|*I*|), where the first ∑ is the sum over all reflections, and the second ∑ is the sum over all measurements of a given reflection, with *I*_i_ being the *i*th measurement of the intensity of the reflection and 〈*I*〉 the average intensity of that reflection.

^*b*^*R*work/*R*free = ∑(|*F*_o_| − 〈|*F*_c_|〉)/∑|*F*_o_|, where 〈|*F*_c_|〉 is the expectation of |*F*_*c*_| under the probability model used to define the likelihood function. The sum is over all reflections.

^*c*^Calculated by use of the MolProbity program.

Highest resolution shell is shown in parenthesis.

Like in the crystals of the isolated NS3 helicase domain [44], the two molecules of NS4A_37_NS3 present in the asymmetric unit (AU) are related by a local 2-fold axis around the helicase domains in which D1 and D3 make a head-to-tail crystallographic dimer interaction (Fig 1B, left panel). Within a pair, one molecule adopts an elongated conformation (Z_max_ = 105 Å) similar to what has been observed for different flavivirus NS3 proteins [45–47] and the other, a more compact conformation (Z_max_ = 69 Å), similar to HCV NS3 [48] depending on the different orientations of the protease domain relative to the helicase domain (Fig 1C). In the AU, both molecules are displaying no visible electron density at the interdomain region of the protein (residues 195 to 204 in the compact conformation and residues 196 to 201 in the elongated conformation). We had, however, an unambiguous choice for the protease/helicase pairs after measuring the distances between the domains. Also, SDS-polyacrylamide gel electrophoresis analysis confirmed that the protein recovered from our crystals remained intact (S2 Fig).

### The structure of NS4A_37_NS3 reveals surface interactions between the NS4A-kink region and the NS3 protease domain

The overall shape of the pestiviral NS4A_37_NS3 is very similar to its counterparts from the *Flavivirus* and *Hepacivirus* genera with two separate globular domains, representing the protease and helicase, linked by a short flexible interdomain region that explains the different relative orientations of the two domains in our structures (Fig 1 and S3 Fig).

In the context of full-length NS4A_37_NS3, the NS3hel domain shows no structural differences and the same flexibility of the helicase domain 2 with respect to the isolated domain [44]. The CSFV NS3pro domain, which comprises 192 residues, shares only 3% of sequence identity among the NS3 proteases within the *Flaviviridae* family (S4A Fig). It has the canonical trypsin-like fold containing two β-barrels, with the conserved Asp-His-Ser catalytic triad located in a cleft between them (S4B Fig). Despite the low percentage of sequence identity, pestiviral NS3pro superposed well to HCV NS3pro with a Root Square Mean Deviation (RSMD) of 1.39 Å and to Dengue virus 4 (DENV4) NS3pro with an RMSD of 2.37 Å, for 129 residues of 178 and 158 Cα positions, respectively, highlighting their close structural similarity (S4B Fig).

**Fig 2:**
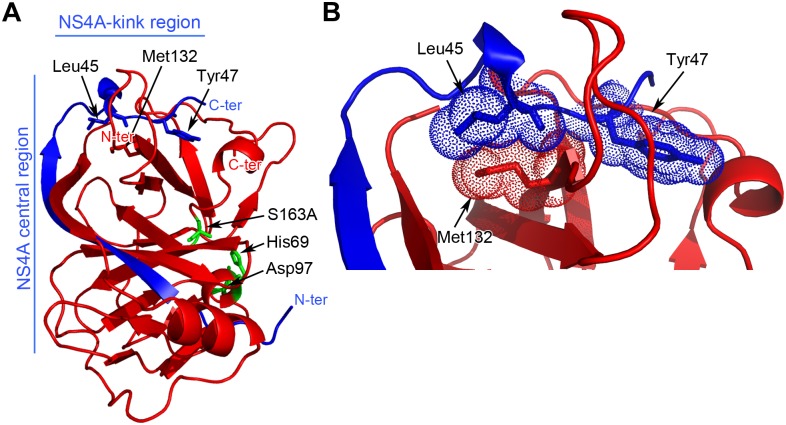
NS3 residue 132 is located at the surface of NS3 at the interaction interface with the NS4A-kink region. (A) Ribbon diagram of the CSFV NS3 protease domain (red) in complex with its cofactor NS4A (blue). NS3 residue Met132, critical for NS2-3-independent morphogenesis, is located at the NS3/4A-kink interface. NS4A residues Leu45 and Tyr47 of the NS4A-kink region and the N- and C-termini for NS3 and NS4A are indicated. Active site residues of NS3pro are indicated (green). (B) Zoom view of the hydrophobic interface of the NS3/NS4A-kink region highlighting residues 3/Met132, 4A/Leu45 and 4A/Tyr47, with spheres representation.

Like in hepaciviral- and flaviviral proteases, the pestiviral NS3 protease domain seems also to be stabilized by the insertion of a β-sheet from NS4A (residues 21 to 40) (Fig 2A). Most interestingly, the C-terminal region of the pestivirus NS4A, namely residues 41 to 49, hereafter referred to as "kink region", is interacting with a hydrophobic patch on the surface of NS3pro. For HCV, structural data of the equivalent region of NS4A in complex with NS3 are so far not available.

Recently, a critical role in pestivirus morphogenesis was assigned to amino acid exchange 3/V132A at position 132 in the NS3 protease domain of BVDV [43]. Interestingly, residue 132, a methionine in the closely related CSFV (valine in BVDV) and most other pestivirus strains [43] is located on the NS3 protease surface and makes hydrophobic contacts mainly with L45 of the NS4A-kink region (Fig 2B). We thus hypothesized that the gain of function mutation identified in BVDV NS3 favors a conformational state required for virion morphogenesis in which the interaction between NS3 and the NS4A-kink region is destabilized. To challenge this hypothesis, we developed a protease protection assay to determine if mutations at the NS3/NS4A-kink interface indeed disturb this interaction.

### Mutations at the interface between the NS3 protease domain and the NS4A-kink region destabilize this interaction

**Fig 3:**
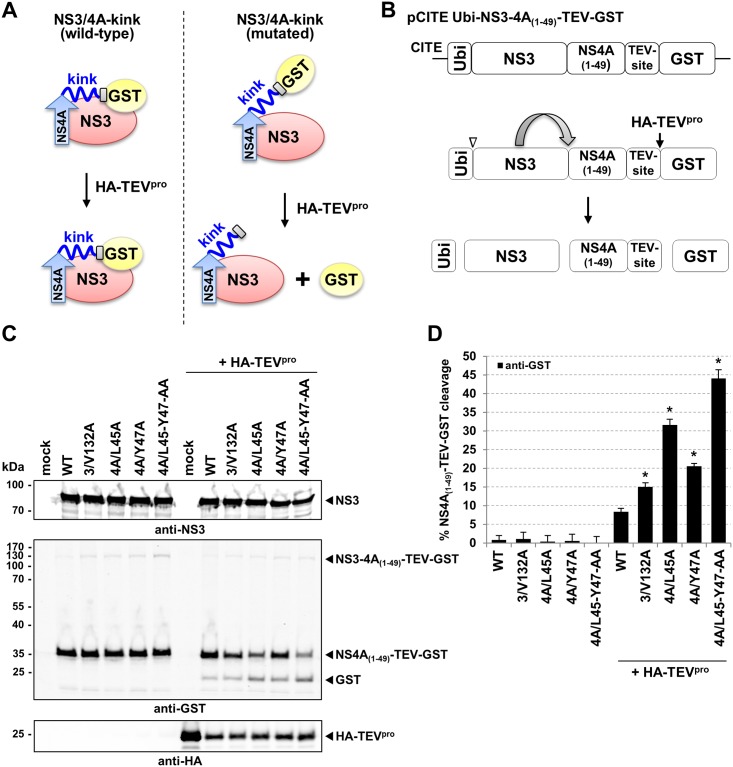
Mutations at the NS3/4A-kink interface expose the C-terminal part of NS4A to TEV^pro^ cleavage. (A) Schematic depiction of the TEV^pro^ protection assay. We hypothesize that wild-type NS3 stably interacts with the NS4A-kink region shielding the TEV cleavage site against proteolysis. Destabilization of the NS3/4A-kink interaction by mutations at the interface would expose the TEV cleavage site to TEV^pro^ -mediated digestion. NS4A (blue) from aa 1–49 was fused to a TEV cleavage site (grey) followed by GST (yellow). NS3 (red), the NS4A-central peptide (arrow) and the NS4A-kink region (kink) are indicated. (B) Scheme of the pCITE Ubi-NS3-4A_(1–49)_-TEV-GST expression construct. Ubiquitin (Ubi) was inserted upstream of full-length NS3 to mediate the generation of an authentic N terminus of NS3. Individual cleavage products generated by cellular ubiquitin-specific proteases (∇), NS3 (↷) and TEV^pro^ (**↓**) are indicated. (C) Western blot analysis of the TEV^pro^ protection assay. Huh7-T7 cells were infected with MVA-T7pol Vaccinia virus followed by co-transfection with the wild-type (WT) pCITE Ubi-NS3-4A_(1–49)_-TEV-GST expression construct or mutant derivatives as indicated above the blot, either without (left part) or with an expression plasmid encoding HA-tagged TEV^pro^ (+ HA-TEV^pro^). Cell lysates prepared 18 h post transfection were analyzed by Western blotting using antibodies directed against NS3, GST and HA-tag. IRDye 800CW-labeled secondary antibodies were applied to allow for quantitative analysis using an Odyssey SA infrared imaging system (*LI-COR*). Molecular mass markers are indicated on the left (kDa). (D) Signals of NS4A_(1–49)_-TEV-GST and GST were quantified and the percentage of NS4A_(1–49)_-TEV-GST cleavage was calculated. Mean values and standard deviations of three experiments are depicted. Groups differed significantly (Kruskal-Wallis test, P < 0.05), and asterisks indicate statistically significant pairwise differences (Mann-Whitney test, P < 0.05) compared to wild-type (WT).

In order to address the hypothesis that mutation 3/V132A destabilizes the NS3/4A-kink interaction, we established a TEV-protease (TEV^pro^) protection assay illustrated in Fig 3A and 3B. We assumed that in the wild-type context a tight association between the NS3 protease surface and the NS4A-kink region would protect the C-terminal NS4A region from a proteolytic attack while destabilizing alanine mutations at the NS3/4A-kink interface should result in higher accessibility to proteolytic cleavage (Fig 3A).

The basic expression construct of the developed assay, pCITE Ubi-NS3-4A_(1–49)_–TEV-GST (Fig 3B), encodes for one ubiquitin monomer, full-length NS3, NS4A (aa 1–49) including the putative end of the NS4-kink region, followed by a TEV cleavage site and GST (Fig 3A and 3B). The ubiquitin moiety mediates the generation of the authentic N terminus of NS3 by cellular ubiquitin hydrolases. Processing by the NS3 serine protease *in cis* is releasing NS4A_(1–49)_-TEV-GST (Fig 3B). Cleavage at the TEV cleavage site, which liberates GST, is achieved by co-expression of a HA-tagged TEV^pro^-encoded by pGEM-T HA-TEV^pro^. In addition to WT pCITE Ubi-NS3-4A_(1–49)_–TEV-GST derivatives encoding alanine mutations at the NS3/4A-kink interface were generated. Besides the gain of function mutation 3/V132A in the NS3 protease domain, derivatives with mutations in the NS4A-kink region (4A/L45A, 4A/Y47A, and 4A/L45-Y47-AA) were established.

Vaccinia virus MVA-T7pol-mediated expression of the described constructs in Huh7-T7 cells resulted in the expected processing pattern for all constructs, i.e. authentic NS3 and NS4A_(1–49)_-TEV-GST demonstrating the functionality of the NS3-4A protease derivatives (Fig 3C, left lanes). Moreover, free GST was not detected providing evidence that GST is not liberated from NS4A_(1–49)_-TEV-GST in absence of HA-TEV^pro^. Upon co-transfection of pCITE Ubi-NS3-4A_(1–49)_–TEV-GST and pGEM-T HA-TEV^pro^, free GST could be detected. Importantly, NS4A_(1–49)_-TEV-GST cleavage did occur to an individual degree (Fig 3C, right lanes). While the lowest level of TEV^pro^-mediated cleavage was detected for wild-type, single mutations introduced either into NS3 (3/V132A) or into the NS4A-kink region (4A/L45A, and 4A/47A) resulted in an increase of NS4A_(1–49)_-TEV-GST cleavage which is indicative of a more accessible TEV cleavage site within the mutant NS3/4A complexes supporting our initial hypothesis. Even more efficient cleavage was detected for the derivative encoding the double mutation in the NS4A-kink region (4A/L45-Y47-AA) (Fig 3C). Quantification of the cleavage efficacies revealed for the wild-type construct a cleavage rate of about 8%, while for the single mutants (3/V132A, 4A/L45A and 4A/Y47A) cleavage rates between 15–32% were observed. The highest cleavage rate of about 44% was determined for the NS4A double mutant 4A/L45-Y47-AA (Fig 3D).

These results show that the gain of function mutation 3/V132A as well as the mutations in the NS4A-kink region expose the TEV cleavage site suggesting a destabilization of the NS3/4A-kink interaction. An interesting and attractive hypothesis that arises from these results is that the protein-protein interaction at the NS3/4A-kink region interface might dictate the formation of distinct conformational states (more open or more closed) of the NS3/4A complex with the potential to serve different functions in the viral life cycle.

### Single mutations at the NS3/4A-kink interface allow for polyprotein processing and RNA replication

**Fig 4:**
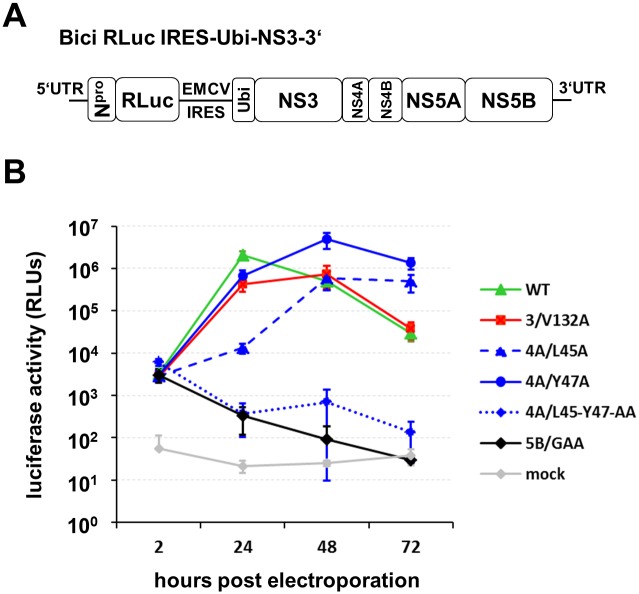
Single mutations at the NS3/4A-kink interface allow for RNA replication. (A) Scheme of the bicistronic replicon Bici RLuc IRES-Ubi-NS3-3’ used to analyze the RNA replication efficacy. The first open reading frame (ORF) encodes for N^pro^ followed by the *Renilla* luciferase and the second ORF for the viral replicase proteins NS3-5B. Ubiquitin (Ubi) was inserted to generate the authentic N terminus of NS3. (B) MDBK cells were electroporated with 1 μg of the corresponding RNA transcript and luciferase activity was determined at 2 h, 24 h, 48 h and 72 h post electroporation. Mean values and standard deviations of four experiments are shown. Mock: electroporated without RNA; 5B/GAA: RNA replication-deficient NS5B mutant; WT: wild-type; RLUs: relative light units; RLuc: *Renilla* luciferase.

Since NS3-4A is the viral main protease, modulation of the interaction at the NS3/4A-kink interface might cause defects in polyprotein processing which finally could result in replication defects. Accordingly, we tested the mutations at the NS3/4A-kink interface first for effects on polyprotein processing and in a second set of experiments for their impact on RNA replication (S5 Fig and Fig 4). Since some mutants might not be replication competent, the replication-independent Vaccinia virus T7 RNA polymerase expression system was used to drive BVDV polyprotein expression in Huh7-T7 cells transfected with WT or mutant BVDV pT7-DI-388 cDNA clones (S5A Fig, [19]). Our analyses revealed authentic polyprotein processing for all NS3-4A mutants (3/V132A, 4A/L45A, 4A/Y47A, and 4A/L45-Y47-AA), as there were no major differences in the accumulation of NS3, NS4A, NS5A or NS5B detectable when compared to wild-type (S5B and S5C Fig). In addition, no differences regarding protein precursors NS3-4A and NS4A-4B were recognized. Since the NS4A mutations interfered with anti-NS4A detection by Western blot, co-radioimmunoprecipitation (Co-RIP) of the respective NS3/4A complex was performed (S5C Fig). No differences regarding polyprotein processing of the individual NS3-4A variants were detected (S5C Fig).

In order to test the effect of the individual mutations on RNA replication efficiency the bicistronic replicon Bici RLuc IRES-Ubi-NS3-3’ was applied, which encodes *Renilla* luciferase in the 5´ open reading frame (ORF) and ubiquitin (Ubi) followed by NS3-5B in the 3´ ORF (Fig 4A). Upon electroporation of the *in vitro* transcribed wild-type replicon RNA and its derivatives, luciferase activity was determined at 2 h, 24 h, 48 h and 72 h post electroporation (pe) (Fig 4B). The luciferase values obtained at 2 h pe represent input RNA translation and demonstrated equal electroporation efficiencies for all transcripts. For the wild-type replicon 2 x 10^6^ RLUs were measured at 24 h pe, followed by a decline at 48 h and 72 h due to a cytopathic effect [19, 43]. The replicon with the 3/V132A mutation showed a slightly reduced RNA replication profile compared to wild-type. In contrast, derivatives encoding single mutations in the NS4A-kink region (4A/L45A and 4A/Y47A) exhibited delays in RNA replication. While the NS4A mutant 4A/Y47A displayed a delay of 24 h compared to wild-type (Fig 4B), mutant 4A/L45A was even more affected: its RNA replication efficacy was 100-fold lower than wild-type 24 h pe with a maximum of 5 x 10^5^ RLUs at 48 h pe (Fig 4B). Interestingly, the introduction of the double mutation 4A/L45-Y47-AA in the NS4A-kink region completely abolished RNA replication (Fig 4B). The observed differences in the RNA replication pattern of the mutants in comparison to the wild-type were further confirmed by statistical analyses (S6 Fig).

Taken together, the single or a double mutations introduced at the NS3/4A-kink interface have no obvious negative effects on polyprotein processing. However, those mutations influence RNA replication to different degrees: mutants that affect the NS3/4A-kink interaction in the TEV^pro^ protection assay only moderately (3/V132A, 4A/L45A and 4A/Y47A, Fig 3D) still allow for RNA replication (Fig 4B). The double mutant that was most affected in the NS3/4A-kink interaction was no longer capable of RNA replication and thus not suited for further experiments in the replicative context.

### The gain of function mutation 3/V132A can be functionally substituted by single mutations in the NS4A-kink region in virion morphogenesis

**Fig 5:**
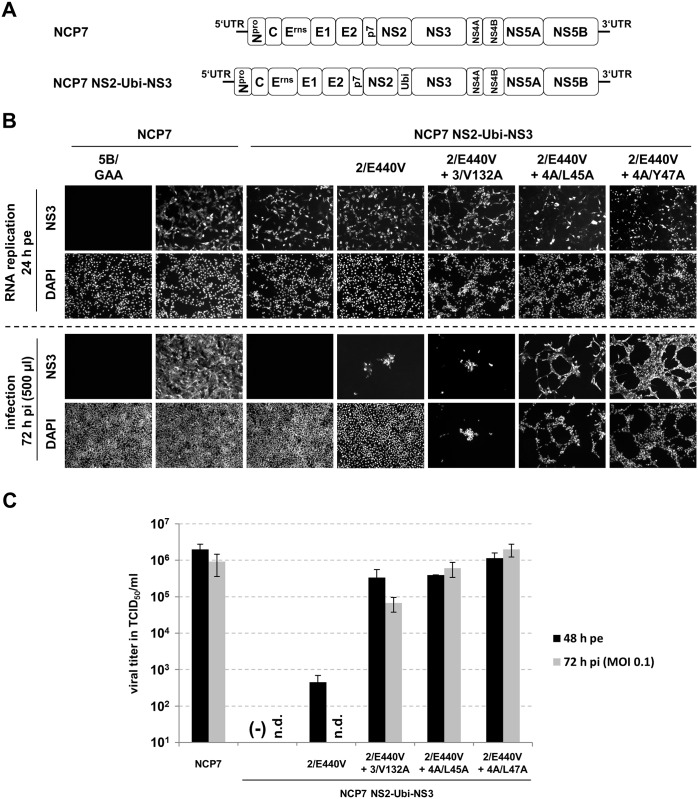
The gain of function mutation 3/V132A can be functionally substituted by the single mutations 4A/L45A or 4A/Y47A in NS2-3-independent virion morphogenesis. (A) Schematic depiction of the pestiviral genomes. NS2-3-independent virion morphogenesis was analyzed using NCP7 in which NS2 is separated from NS3 by Ubi. (B) Top, RNA replication: MDBK cells were electroporated with 1 μg of the respective *in vitro* transcribed RNA. RNA replication was indirectly analyzed by immunofluorescence (IF) of NS3 at 24 h pe. Bottom, infection: Naïve MDBK cells were inoculated with 500 μl of the supernatants harvested at 48 h pe. At 72 h post inoculation, infected cells were detected by anti-NS3 IF. (C) Viral titer analyses. Cell culture supernatants collected at 48 h pe or 72 h pi (MOI 0.1) were analyzed for infectious virus by limited dilution assay (TCID_50_/ml). Mean values and standard deviations of viral titers of three experiments are depicted. Mutations in NS2 (2/E440V), NS3 (3/V132A) and NS4A (4A/L45A and 4A/Y47A) are indicated. UTR: untranslated region; 5B/GAA: RNA replication-deficient NS5B mutant; (-) no virus detected; n.d.: not determined.

A moderate modulation of the NS3/4A-kink interaction could be achieved by either the previously described gain of function mutation in NS3 (3/V132A) or by single amino acid exchanges in the NS4A-kink region (4A/L45A and 4A/Y47A) (Fig 3). These mutations still allowed for RNA replication which enabled us to test whether the NS4A mutations 4A/L45A and 4A/Y47A could functionally substitute the NS3 mutation V132A in NS2-3-independent virion morphogenesis in the context of variants of BVDV strain NCP7 [43]. To this end, NCP7 NS2-Ubi-NS3 (2/E440V + 3/V132A), encoding both previously described gain of function mutations, was compared with NCP7 NS2-Ubi-NS3 (2/E440V + 4A/L45A) and NCP7 NS2-Ubi-NS3 (2/E440V + 4A/Y47A) (Fig 5). Full-length NCP7 NS2-Ubi-NS3 is unable to produce viral progeny and served as negative control for NS2-3-independent virion morphogenesis. In addition, the NCP7 NS2-Ubi-NS3 variant carrying only the 2/E440V mutation was tested to exemplify the importance of the mutation 3/V132A to allow for efficient NS2-3-independent particle formation, as described previously [43]. RNA transcripts of the non-cytopathic BVDV NCP7 wild-type (NCP7) and the replication-defective mutant (NCP7 5B/GAA) were electroporated into MDBK cells and served as positive or negative control, respectively; RNA replication was monitored indirectly *via* detection of NS3 at 24 h pe by an immunofluorescence assay (Fig 5B, Top panel, RNA replication).

MDBK cells electroporated with RNA transcripts of pNCP7 NS2-Ubi-NS3 and its derivatives containing mutations (2/E440V + 3/V132A), (2/E440V + 4A/L45A) or (2/E440V + 4A/Y47A) were positive for NS3 at 24 h pe (Fig 5B, Top panel) demonstrating that the combinations of mutations 2/E440V with either 4A/L45A or with 4A/Y47A allow for RNA replication in the context of a full-length virus.

To investigate the formation of infectious progeny, cell culture supernatants of the electroporated cells harvested at 48 h pe were used to inoculate naïve MDBK cells which were analyzed 72 h later for the presence of NS3 to monitor infection (Fig 5B, Bottom panel). The replication-defective NS5B mutant 5B/GAA and BVDV NCP7 wild-type served as negative and positive controls, respectively. As expected, supernatants of cells electroporated with NCP7 NS2-Ubi-NS3 RNA did not contain infectious virus. The NCP7 NS2-Ubi-NS3 variant encoding only for the mutation 2/E440V in NS2 was able to produce infectious virus to a very limited amount, as expected from previous studies [43]. When supernatants from cells electroporated with RNAs NCP7 NS2-Ubi-NS3 (2/E440 + 3/V132A) were used to inoculate naïve MDBK cells a high number of NS3-positive cells and a severe cytopathic effect was observed, corroborating the critical role of these mutations for virion morphogenesis [43]. Next we analyzed virion production of virus variants in which mutation 3/V132A was replaced by a single mutation in NS4A. Most importantly, the transfer of supernatants from cells electroporated with RNAs NCP7 NS2-Ubi-NS3 (2/E440V + 4A/L45A) or NCP7 NS2-Ubi-NS3 (2/E440V + 4A/Y47A) to naïve MDBK cells resulted in NS3-positive cells and a cytopathic effect indicating the presence of infectious virus (Fig 5B, Bottom panel–compare NS3 and DAPI stain).

The amounts of infectious viruses within supernatants collected at 48 h pe or 72 h after a defined infection of naïve MDBK cells at MOI 0.1 were determined in TCID_50_/ml (Fig 5C). As expected, the NCP7 NS2-Ubi-NS3 virus was not able to generate viral progeny. In accordance to previous studies [43], the virus encoding 2/E440V together with 3/V132A showed high viral titers of 3.4 x 10^5^ (48 h pe) or 6.7 x 10^4^ after defined infection at MOI 0.1. As expected, the derivative of NCP7 NS2-Ubi-NS3 encoding only NS2/E440V produced low amounts of infectious viruses (4.5 x 10^2^–48 h pe) underlining the importance of the mutation 3/V132A for efficient particle formation in absence of uncleaved NS2-3 [43]. Importantly, viruses encoding mutations in the NS4A-kink region (NCP7 NS2-Ubi-NS3 (2/E440V + 4A/L45A); NCP7 NS2-Ubi-NS3 (2/E440V + 4A/Y47A)) instead of 3/V132A were also able to reach high titers at 48 h pe (4A/L45A: 4.0 x 10^5^; 4A/Y47A: 1.1 x 10^6^) or after defined infection at MOI 0.1 (4A/L45A: 6.0 x 10^5^; 4A/Y47A: 2.0 x 10^6^) (Fig 5C).

In sum, these results demonstrate a highly efficient virus production for all BVDV NCP7 NS2-Ubi-NS3 derivatives with single mutations at NS3/NS4A-kink interface when combined with the mutation 2/E440V in NS2.

### The replication-deficient NS3/4A double mutant 4A/L45-Y47-AA is functional in viral packaging when supplied *in trans*

The data obtained so far were in line with a regulatory role of the stability of the NS3/4A-kink interaction for the decision whether a NS3/4A complex is functional in RNA replication and/or virion morphogenesis. In this context the double mutation 4A/L45-Y47-AA which is severely destabilizing the NS3/4A-kink interaction (Fig 3) was of special interest since it leads to a NS3/4A complex that is not able to support viral RNA replication (Fig 4B). However, it was still conceivable that this NS3/4A complex is capable of supporting virion morphogenesis.

**Fig 6:**
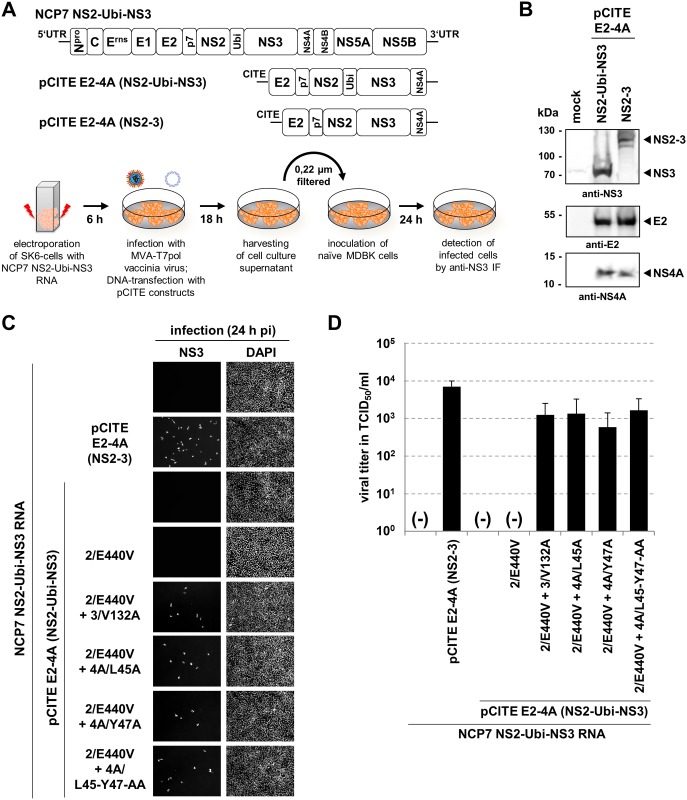
A *trans*-complementation assay revealed that the replication-deficient NS3/4A double mutant 4A/L45-Y47-AA is functional in viral packaging. (A) Top: Schematic depiction of BVDV-1 NCP7 NS2-Ubi-NS3 and the pCITE expression constructs. Viral structural proteins (C, E^rns^, E1, E2), and NS-proteins N^pro^, p7-NS5B are indicated. Bottom: Illustration of the *trans*-complementation assay. SK6-cells were electroporated with 1 μg of *in vitro* transcribed NCP7 NS2-Ubi-NS3 RNA. 6 hours pe, cells were infected with MVA-T7pol vaccinia virus and subsequently transfected with 8 μg of the pCITE expression constructs. After 18 hours, supernatants were harvested and filtered. Subsequently, naïve MDBK cells were inoculated with the respective supernatant. 24 h later cells were monitored for infected cells by anti-NS3 IF. UTR: untranslated region; Ubi: ubiquitin; CITE: EMCV-IRES. (B) MVA-T7pol vaccinia virus based expression of E2-4A polyprotein derivatives analyzed by Western blot with the indicated antibodies. Molecular mass markers are indicated on the left (kDa). (C) *Trans*-complementation assay. MDBK cells electroporated with NCP7 NS2-Ubi-NS3 RNA were transfected with the indicated plasmids for MVA-T7 based protein expression. At 24 h post transfection the supernatants were used to inoculate naïve MDBK cells which were analyzed 24 h later by anti-NS3 IF. One representative assay out of three performed is shown. (D) Viral titers are given in TCID_50_/ml. Mean values and standard deviations of three performed experiments are depicted. Mutations in NS2 (2/E440V), NS3 (V132A), and NS4A (4A/L45A, 4A/Y47A and 4A/L45-Y47-AA) are indicated. NS2-3: uncleaved NS2-3 protein; (-): no NS3-positive cells were detected.

Since it was not possible to investigate the functionality of this replication-deficient mutant in the context of a recombinant pestiviral genome a *trans*-complementation assay was established analogous to an approach published by Moulin *et al*. [29] (Fig 6A). To rescue virion formation of the packaging incompetent NCP7 NS2-Ubi-NS3, MVA-T7pol-mediated plasmid-based expression of E2-4A variants was employed (Fig 6A). SK6-cells were chosen since Vaccinia virus-based gene expression as well as DNA transfection is highly inefficient in MDBK cells. Expression of the wild-type E2-4A polyprotein from pCITE E2-4A (NS2-3) was expected to rescue virion formation and served as positive control; expression of an E2-4A derivative with ubiquitin between NS2 and NS3 (NS2-Ubi-NS3) served as negative control (Fig 6A). Western blot analysis verified the generation of the uncleaved NS2-3 from pCITE E2-4A (NS2-3) and processed NS3 but no uncleaved NS2-Ubi-NS3 precursor protein upon expression of pCITE E2-4A (NS2-Ubi-NS3) (Fig 6B). In addition, correct processing of E2 and NS4A was observed for both constructs (Fig 6B).

The mutations at the NS3/4A-kink interface (3/V132A, 4A/L45A, 4A/Y47A or 4A/L45-Y47-AA), each combined with mutation 2/E440V, were introduced into pCITE E2-4A (NS2-Ubi-NS3) and tested for their functionality in viral *trans*-packaging.

To this end, packaging incompetent full-length NCP7 NS2-Ubi-NS3 RNA was electroporated into SK6-cells, followed by Vaccinia MVA-T7pol-based protein expression from the individual pCITE E2-4A (NS2-Ubi-NS3) constructs. At 24 h post transfection, cell culture supernatants were filtered and used to inoculate naïve MDBK cells (Fig 6C). The anti-NS3 IF assay revealed infected cells and therefore successful rescue of virion formation by the positive control E2-4A (NS2-3) but not for E2-4A (NS2-Ubi-NS3) (Fig 6C). Additional *trans*-complementation experiments with the derivative E2-4A (NS2-Ubi-NS3) encoding only 2/E440V resulted in no viral rescue. In order to analyze for RNA-recombination events that may result in the formation of infectious viruses, we analyzed for infected cells (NS3-positive cells) also at 96 h after inoculation (S7 Fig). Neither single NS3-positive cells nor infected foci were detected (S7B Fig). While the absence of individual NS3-positive cells is most likely due to the cytopathic effect caused by the replication of the NCP7 NS2-Ubi-NS3 RNA the absence of antigen-positive foci excluded the presence of infectious virus generated by RNA recombination. Importantly, all tested E2-4A (NS2-Ubi-NS3) derivatives with mutation 2/E440V in combination with single alanine exchanges either in the NS3 protease domain (3/V132A) or the NS4A-kink region (4A/L45A and 4A/Y47A) were able to rescue virion formation (Fig 6C). Strikingly, also expression of E2-4A (NS2-Ubi-NS3) 2/E440V with the double mutation (4A/L45-Y47-AA) in NS4A allowed virion morphogenesis.

Quantitative analysis of supernatants revealed a mean virus titer of 7.1 x 10^3^ TCID_50_/ml when E2-4A (NS2-3) was supplied *in trans*. Importantly, all constructs which encode E2-4A (NS2-Ubi-NS3) variants combining 2/E440V with 3/V132A (1.3 x 10^3^ TCID_50_/ml), 4A/L45A (1.4 x 10^3^ TCID_50_/ml), 4A/Y47A (5.9 x 10^2^ TCID_50_/ml) or 4A/L45-Y47-AA (1.7 x 10^3^ TCID_50_/ml) were able to rescue virion morphogenesis of NCP7 NS2-Ubi-NS3 (Fig 6C and 6D).

The results of the *trans*-complementation lead to the intriguing conclusion that the NS4A double mutant 4A/L45-Y47-AA, while interfering with viral RNA replication, is still functional in virion morphogenesis.

### The NS2 moiety modulates the NS3/4A-kink interaction within the NS2-3/4A complex

**Fig 7:**
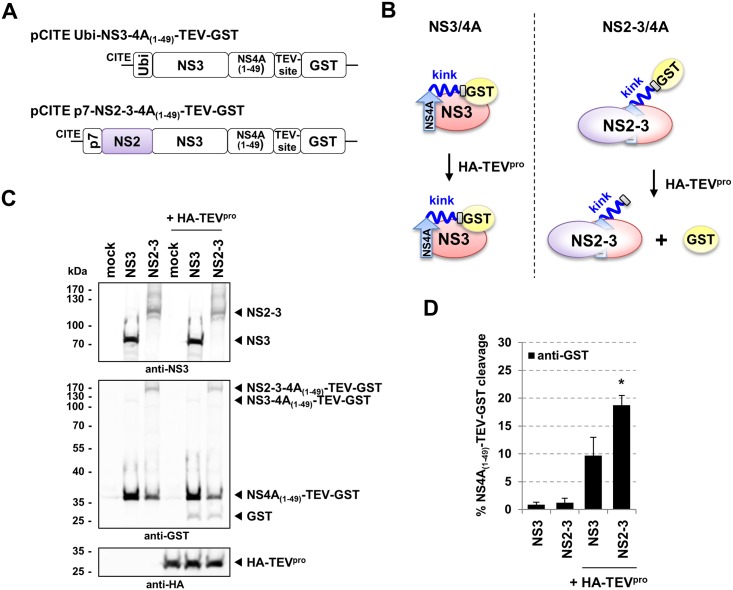
The presence of NS2 contributes to a decreased NS3/4A-kink interaction in the NS2-3/4A complex. (A) Schematic depiction of the pCITE constructs. pCITE Ubi-NS3-4A_(1–49)_-TEV-GST is described in Fig 3. pCITE p7-NS2-3-4A_(1–49)_-TEV-GST encodes full-length NS2-3; p7 sequence was introduced upstream of NS2 (purple) to allow for correct processing of the N terminus of NS2. (B) Scheme of the TEV^pro^ protection assay. In the NS3/4A complex, the NS4A-kink region (kink) interacts with NS3 (red) thereby reducing TEV^pro^ cleavage (left panel). The presence of uncleaved NS2-3 exposes the TEV cleavage site and allows for increased TEV^pro^-mediated cleavage (right panel). NS4A (blue), TEV cleavage site (grey), GST (yellow) and the NS4A-central peptide (arrow) are indicated. (C) Western blot analysis of the TEV^pro^ protection assay. Huh7-T7 cells were infected with MVA-T7pol vaccinia virus and incubated for 1 h at 37°C followed by co-transfection with the expression constructs indicated above, either without (left part) or with an TEV-protease expression plasmid (+HA-TEV^pro^). Western blot analyses with anti-NS3, anti-GST and anti-HA are shown; for details see legend of Fig 3. (D) Quantification of NS4A_(1–49)_-TEV-GST cleavage. Mean values and standard deviations of three independent experiments are depicted. The significance of the differences between the NS3 and the NS2-3 variant was calculated by Student’s t-test (* P = 0.013).

A destabilization of the NS3/4A-kink interaction appears to be a prerequisite to enable the NS3/4A complex to be active in virion morphogenesis. However, in wild-type pestiviruses NS2-3/4A but not NS3/4A is employed in packaging. Accordingly, the mutations at the NS3/4A-kink interface represent functional surrogates for the presence of NS2 in the NS2-3/4A complex. In consequence, we hypothesized that in the NS2-3/4A complex the NS2 protein moiety should have an effect similar to the one observed for the mutations at the NS3/NS4A interaction surface, namely the destabilization of the NS3/4A-kink interaction. To test our hypothesis we applied the TEV^pro^ protection assay by which we compared pCITE Ubi-NS3-4A_(1–49)_–TEV-GST with pCITE p7-NS2-3-4A_(1–49)_–TEV-GST (Fig 7A and 7B). The p7 gene was included in pCITE p7-NS2-3-4A_(1–49)_–TEV-GST since p7 contains a leader sequence to properly insert NS2 into the ER membrane and to allow the generation of its authentic N terminus by cellular signal peptidase [49]. As described in Fig 3 we determined the levels of TEV cleavage by Western blot and subsequent quantification (Fig 7C and 7D). When analyzed in parallel, a cleavage rate of 10% was observed for pCITE Ubi-NS3-4A_(1–49)_–TEV-GST while 19% were obtained for pCITE p7-NS2-3-4A_(1–49)_–TEV-GST (Fig 7D). Thus, the TEV^pro^ protection assay indicates that the NS2-3/4A complex adopts a conformation more accessible to TEV^pro^ cleavage when compared to the NS3/4A complex.

Taken together, these results support the hypothesis that the modulation of the NS3/4A-kink interaction either by mutation at the NS3/4A-kink interface or by the presence of NS2 in the NS2-3/4A complex lead to a less compact conformation required for virion morphogenesis.

## Discussion

Due to their compact genome size viruses encode a severely limited number of gene products which therefore often exert multiple functions. Along these lines, the nonstructural proteins of the members of the *Flaviviridae* family have key functions during RNA replication and in the assembly of infectious particles [21]. In addition, like most positive-strand RNA viruses, pestiviruses use regulated polyprotein processing by viral and cellular proteases as means to temporal and spatial regulate protein activity [3]. An excellent example is the observation that in the pestiviral life cycle, RNA replication and virion morphogenesis are highly regulated processes both depending on different protein complexes which derive from the NS2-3-4A region of the polyprotein: while the NS3/4A complex is essential for RNA replication and cannot be functionally replaced by NS2-3/4A [6, 18], NS2-3/4A is indispensable for pestiviral particle formation but is not active in RNA replication [28, 29]. NS2-3 cleavage by the NS2 autoprotease is temporally restricted to the early time points of infection due to its dependency on a cellular cofactor available only in limiting amounts [6, 37]. This leads to a temporal gradient for the formation of NS3/4A vs. NS2-3/4A in pestivirus infected cells. In contrast to pestiviruses, the closely related HCV does not depend on uncleaved NS2-NS3 for infectious virion formation at least in cell culture [26, 41] and no major amounts of uncleaved NS2-NS3 can be detected in HCV-infected cells. Thus, HCV and pestiviruses show a major difference with regard to their dependency on uncleaved NS2-3 for the formation of infectious progeny.

Interestingly, pestiviruses can be adapted by two amino acid exchanges, one in NS2 and the other one in NS3, to virion morphogenesis without the requirement for uncleaved NS2-3 and thus can carry out virion assembly with strong parallels to HCV [42, 43]. The NS3 mutation 3/V132A resulting in a gain of function of the NS3/4A complex in virion morphogenesis was of central importance for this study. In view of the critical role of the NS3/4A protein complex in the switch between RNA replication and virion assembly, the determination of the structure of a single-chain CSFV NS3 serine protease-NS4A complex provided the basis for structure-guided functional analyses.

The CSFV full-length NS3 structure in complex with its cofactor NS4A shows that the central region of NS4A forms a β-sheet which intercalates into the N-terminal β-barrel of the NS3 serine protease domain, similar to the results reported for HCV NS3 when co-crystallized with a NS4A cofactor peptide (Fig 1) [11]. However, a question not answered by the available HCV NS3/4A structures was whether its NS4A-kink region undergoes additional interactions with the NS3 surface [11, 50]. The structure of the CSFV single-chain NS3/4A complex reported in this study provided insights into this aspect (Fig 2A and 2B). We found that residue M132 maps to the surface of the NS3 protease domain and makes hydrophobic interactions mainly with residues L45 and Y47 of the NS4A-kink region through specific and main chain interactions, respectively (Fig 2B). Our data support the idea that these hydrophobic contacts are critical for a compact conformation of NS3/4A required when serving as a component of the replicase complex. Since an exchange of this residue to Ala (3/V132A) is critical for the gain of function in NS2-3-independent virion formation we speculated that the modulation of this interaction between NS3 and the NS4A-kink region could be the underlying molecular principle. We hypothesized that a destabilization of this interaction (as introduced by e.g. 3/V132A, 4A/L45A or 4A/Y47A) would lead to a more open conformation of the NS3/4A complex required for its function, together with NS2, in NS2-3-independent virion assembly. To follow this hypothesis a method was required to determine the effect of individual mutations on the interaction at the NS3/4A-kink interface. Since the central domain of NS4A stably intercalates into the NS3 protease domain a dissociation of both proteins was not to be expected as a consequence of a weakened NS3/4A-kink interaction and thus could not serve as read out. Therefore, a TEV^pro^ protection assay was established (Fig 3). It was assumed that a more flexible or open conformation of the NS3/4A-kink region will result in a higher accessibility of the TEV cleavage site to TEV^pro^ (Fig 3A and 3B). As proposed, the accessibility at the TEV cleavage site was elevated for all NS3/4A variants with mutations at the NS3/4A-kink interaction surface when compared to wild-type (Fig 3C and 3D). This was in agreement with a more open or more flexible conformation of the mutant NS3/4A protein complexes. Interestingly, the degree of TEV^pro^ cleavage for the individual mutants did inversely correlate with their replication fitness (Fig 4). The previously selected NS3 mutant 3/V132A displayed a moderately increased TEV cleavage rate and RNA replication was slightly reduced when compared to wild-type. Mutant 4A/L45A, which allowed for a more efficient TEV^pro^ cleavage compared to 4A/Y47A showed lower replication fitness than 4A/Y47A. The NS4A double mutant 4A/L45-Y47-AA displayed the highest TEV^pro^ cleavage and was found incapable of RNA replication (compare Figs 3D and 4B). Interestingly, the C-terminal region of HCV NS4A, which is significantly shorter than the one of pestiviruses, was also shown to be critically involved in replicase assembly. In this region of HCV NS4A, two residues (Y45 and F48) were found to inhibit RNA replication in different genotypes when mutated to alanine [13, 14]. While structural data about this part of the HCV NS3/4A complex is still missing, these residues could be critically involved in an interaction of the C-terminal NS4A region with NS3, especially since the replication defects of several NS4A mutants could be suppressed by second site mutations on the NS3 surface [13] or in NS4B [14]. Recently, a hydrophobic patch on the surface of the HCV NS3 protease domain was identified as a critical determinant for replicase assembly [51]. Future studies have to reveal whether this hydrophobic area on the HCV NS3 surface is involved in interactions with the C-terminal domain of NS4A. Overall, replication studies within different HCV genotypes indicate that genetic interactions between NS3, NS4A, and NS4B contribute to replicase assembly and genome replication [13, 14, 33, 52, 53].

The observation that the replication-deficient BVDV NS4A double mutant retained full NS3-4A protease activity indicates that the respective amino acid exchanges are not interfering with proper folding of the NS3/4A complex (Fig 4 and S5 Fig). This assumption was further corroborated by the fact that the NS4A double mutant was still capable of forming NS3/4A complexes that are active in virion morphogenesis (Fig 6). Moulin *et al*. had already shown that mature NS4A is required in addition to NS2-3 for virion formation and that NS4A fused to the C-terminus of NS3 is not functional [29]. For HCV a role of the C-terminal part of NS4A in viral assembly has been described [14]. Accordingly, this NS4A region is involved in RNA replication and virion morphogenesis also for HCV and thus might represent a switch between these processes [13].

Taken together, our data indicate that the modulation of the interaction between NS3 and the NS4A-kink region represents a way to control the switch between NS3/4A complexes functional either in RNA replication or virion assembly (Table 2).

**Table 2:** Functionality of NS2-3/4A or NS3/4A complexes in RNA replication and viral packaging.

Protein complex	RNA replication ability	viral packaging *in cis*	viral packaging *in trans*
NS3/4A	+	-	-
NS3/4A (3/V132A)	+	+	+
NS3/4A (4A/L45A)	+	+	+
NS3/4A (4A/Y47A)	+	+	+
NS3/4A (4A/L45-Y47-AA)	-	**n.d.**	+
NS2-3/4A	-	+	+

n.d.: not determined

In this context the role of the gain of function mutation 2/E440V which is also strictly required for virion formation in the absence of uncleaved NS2-3, still remains enigmatic mainly due to the absence of structural information for pestiviral NS2. NS2 residue 440 is located in the C-terminal NS2 protease domain that is residing in the cytoplasm [34]. In previous studies correctly folded NS2 was found critical with respect to NS2-3-independent virion formation, as C-terminal truncations or mutations of the Zn-coordinating residues inhibit particle formation [42]. In the HCV system NS2 also holds a central role in virion assembly by recruiting envelope glycoproteins to the site of assembly and participating in multiple protein-protein interactions with structural and nonstructural proteins that are required for virion morphogenesis [26, 27, 41, 54–56]. Further in depth investigations have to clarify if pestiviral NS2 has a similar role as organizer of the virion assembly pathway.

**Fig 8:**
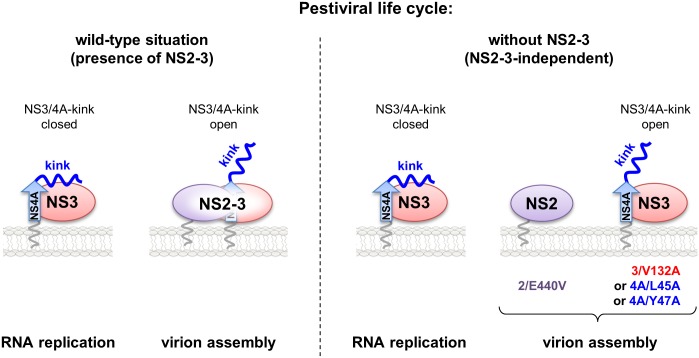
Hypothetical model of pestiviral genome replication and virion morphogenesis in presence and in absence of uncleaved NS2-3. (Left) In the pestiviral wild-type situation the NS3/4A complex is an essential component of the viral replicase while NS2-3/4A is only functional in infectious particle formation. The NS2 moiety in the NS2-3/4A complex induces the open conformation required for its packaging function. NS3 (red), NS2 (purple), uncleaved NS2-3, NS4A (blue), the NS4A central peptide (blue arrow) and the NS4A-kink region (kink). (Right) In the absence of uncleaved NS2-3 pestiviruses require gain of function mutations in NS2 and the NS3/4A complex (NS2-3-independent). The present study indicates that a conformational change at the NS3/4A-kink interface, induced by the indicated mutations, represents a molecular switch between a closed (favored during RNA replication) and an open conformation (favored during viral packaging). Individual mutations in NS2 (E440V), NS3 (V132A) and NS4A (L45A, or Y47A) which allow for NS2-3-independent virion formation are indicated.

The identification of pestivirus mutants which are capable of NS2-3-independent virion morphogenesis was instrumental for this study. However, in wild-type pestiviruses uncleaved NS2-3 is strictly required for virion morphogenesis. By applying the TEV^pro^ protection assay we observed that NS2 in the context of the NS2-3/4A complex recapitulates the effect of the mutations at the NS3/4A interaction surface, namely a modulation of the NS3/4A-kink interaction (Fig 7). Therefore, we propose that NS2 in the NS2-3/4A complex alters the interaction between the NS4A-kink region and the NS3 surface favoring a packaging-competent conformation. This leads to our current working model (Fig 8) in which a closed and a more open conformation of the NS3/4A-kink region allow the assembly of so far not characterized protein complexes critical either for RNA replication or virion morphogenesis, respectively.

This hypothesis is also in line with the fact that the N terminus of NS3 is in close proximity to the NS3/4A-kink interface (Fig 2). In analogy to the pestivirus system, the presence of NS2 at the N terminus of NS3 was also shown to inhibit HCV RNA replication as well as the assembly of a functional RNA-replicase complex indicated by a loss of NS5A hyperphosphorylation [51]. Taken together, these findings support for both virus genera the model that the presence of NS2 in the NS2-3/4A complex leads to a conformation interfering with replicase assembly. However, structural data on uncleaved NS2-3 are absolutely required for further conclusions.

The identification of the basic building blocks for RNA replication and virion morphogenesis will facilitate future work aiming at unraveling the respective pathways. An important next step will be the identification of interaction partners of NS3/4A or NS2-3/4A relevant for either replicase assembly or virion morphogenesis. Furthermore, we expect that similar mechanisms are used by other RNA viruses which face the general challenge of how to address a plethora of functions with a very limited set of proteins.

## Materials and methods

### Expression and purification of NS4A_37_NS3

To express and purify full-length CSFV NS3 (hereafter named NS4A_37_NS3) for crystallization, we used the constructs and the expression and purification methods described in [44]. Briefly, the expressed NS4A_37_NS3 encompasses His-tag, TEV cleavage site (MHHHHHHHENLYFQG), CSFV NS4A amino acids 21 to 57, a GSGS linker, followed by full-length NS3 and the first 8 amino acids of NS4A.

### Crystallization and structure determination

Conditions for crystallization of CSFV NS4A_37_NS3 were found using a Mosquito robot in the format of 96 Greiner plates. Crystals were optimized with a robotized Matrix Maker and Mosquito setups. The best diffracting crystals corresponded to NS4A_37_NS3 carrying a mutation in the protease catalytic site S163A and were grown in sitting drops at 20°C by vapor diffusion under the condition listed in Table 1. For data collection, the crystals were flash cryo-cooled in liquid nitrogen using 20% glycerol as cryoprotectant. Diffraction data were collected at the synchrotron beam line ID23-1 at the European Synchrotron Radiation Facility (Grenoble, France) indexed and processed using XDS [57] and scaled with SCALA [58]. The structure was determined by molecular replacement with PHASER using as a search models the structure of CSFV isolated helicase domain (NS3hel) [44] and an homology model based in the structure of HCV protease generated with Phyre (http://www.sbg.bio.ic.ac.uk/phyre2/html/page.cgi?id=index). The model of CSFV full-length NS3 was subsequently manually modified with Coot [59] and refined with BUSTER-TNT [60].

### Antibodies

For antigen detection, mouse monoclonal antibody 8.12.7 against NS3/NS2-3 [61], anti-E2 (SCR48 6.6.11) (kindly provided by H.-J. Thiel, [Justus-Liebig University, Gießen]) [62], anti-NS4A (GH4A1 [4B7]), anti-NS5A (GLBVD5A1 [11C]), anti-NS5B (GLBVD5B1 [9A]) (kindly provided by T. Rümenapf and B. Lamp [University of Veterinary Medicine, Vienna, Austria]) [43], anti-HA (HA.11 clone 16B12, Covance, New Jersey, USA), anti-GST (New England Biolabs), anti-myc (New England Biolabs) were used. Species specific Cyanogen-3-labeled (Cy3) or peroxidase-coupled (PO) antibodies were obtained from Dianova (Hamburg). Quantitative Western blot analyses were performed using secondary antibody coupled to IRdye-800 obtained from *LI-COR* Biosciences (Lincoln, Nebraska, USA).

### Cells and viruses

Madin-Darby Bovine Kidney (MDBK) cells (American Type Culture Collection—ATCC) were cultivated in minimal essential medium (MEM, Invitrogen) containing 10% horse serum, nonessential amino acids and 1% penicillin/streptomycin (PAA, Pasching, Austria). Swine kidney cells (SK-6) [63] were cultivated in MEM supplemented with 10% fetal bovine serum and 1% penicillin/streptomycin. Huh7-T7 cells [64] were kept in DMEM containing 10% fetal bovine serum gold, 1% penicillin/streptomycin and 125 μg/ml G418 (PAA, Pasching, Austria). All cells were grown at 37°C and 5% CO_2_.

BVDV-1 strain NCP7 was described previously [65, 66]. The original Vaccinia virus modified virus Ankara (MVA)-T7pol stock [67] was generously provided by G. Sutter (LMU, Munich, Germany).

### Plasmids and mutagenesis

BVDV-1 genomes NCP7 [66] and NCP7 NS2-Ubi-NS3 [43] have been described. Tables 3 and 4 summarize all constructs which were used in this study. Further details concerning the generation of the constructs and their properties can be found in the supplementary material.

**Table 3:** BVDV-1 full-length clones used for the analysis of NS2-3-independent virion morphogenesis.

Plasmid name	Feature(s)
pNCP7-388	Encompasses a full-length cDNA genome of BVDV-1 NCP7 [66, 68].
pNCP7-388 NS5B/GAA	Derivative of pNCP7-388. Encodes for an inactive RdRp by mutations GDD to GAA; negative control for RNA replication.
pNCP7 NS2-Ubi-NS3	Derivative of pNCP7-388 encoding the ubiquitin monomer originating from strain Osloss between NS2 and NS3 genes (Ubi) [43].
pNCP7 NS2-Ubi-NS3 (2/E440V + 3/V132A)	Derivative of pNCP7 NS2-Ubi-NS3. Contains the mutations E440V in NS2 (2/E440V) and V132A in NS3 (3/V132A) which have been shown to be essential for NS2-3-independent virion morphogenesis [43].
pNCP7 NS2-Ubi-NS3 (2/E440V + 4A/L45A)	Derivative of pNCP7 NS2-Ubi-NS3. Contains the mutations E440V in NS2 (2/E440V) and L45A in NS4A (4A/L45A).
pNCP7 NS2-Ubi-NS3 (2/E440V + 4A/Y47A)	Derivative of pNCP7 NS2-Ubi-NS3. Contains the mutations E440V in NS2 (2/E440V) and Y47A in NS4A (4A/Y47A).

**Table 4:** Subgenomic expression plasmids which were used in different assays including: TEV^pro^ protection assay, polyprotein processing, RNA replication, *trans*-complementation assay.

Plasmid name and application	Feature(s)
*TEV*^*pro*^ *protection assay*	
pCITE Ubi-NS3-4A_(1–49)_-TEV-GST	Expression construct which encodes for a polyprotein consisting of one monomer of bovine ubiquitin, BVDV-1 full-length NS3, BVDV-1 NS4A from aa 1–49 (P49- the putative end of the 4A-kink region), a cleavage site of the tobacco edge virus (TEV, aa of TEV cleavage site: ENLYFQG), followed by glutathione-S-transferase (GST). Gene expression is under control of a T7-promotor. Further derivatives of pCITE Ubi-NS3-4A_(1–49)_-TEV-GST encoding for individual mutations in either the NS3 protease domain (3/V132A), or in the NS4A-kink region (4A/L45A, 4A/Y47A, or 4A/L45-Y47-AA) were prepared. An NS2-3 variant of pCITE Ubi-NS3-4A_(1-49)_-TEV-GST was created in which ubiquitin was replaced by the gene cassette of p7-NS2 of BVDV-1 strain NCP7.
pGEM-T HA-TEV^pro^	Expression construct that encodes the TEV-protease (TEV^pro^) that contains an HA-tag (aa: YPYDVPDYA) at the N-terminus. Gene expression is under control of a T7-promotor.
*Polyprotein processing*	
pT7-DI-388	Subgenomic replicon based on BVDV pDI9 and strain CP7, encoding viral proteins N^pro^-NS3-5B [18]. In contrast to pDI9 the RNA transcription is under control of a T7-promotor [19]. Further derivatives of pT7-DI-388 encoding for individual mutations in either the NS3 protease domain (3/V132A), or in the NS4A-kink region (4A/L45A, 4A/Y47A, or 4A/L45-Y47-AA) were prepared.
pT7-DI-388 (3/S163A)	Derivative of pT7-DI-388; inactivation of NS3 protease by mutation of the active site serine S163 to alanine.
*RNA replication*	
pBici RLuc IRES-Ubi-NS3-3‘	Bicistronic BVDV reporter gene construct. The 5´ ORF encodes N^pro^ followed by *Renilla* luciferase. The 3´ ORF encodes one monomer of ubiquitin followed by the viral replicase NS3-5B [19]. Further derivatives of pBici RLuc IRES-Ubi-NS3-3‘ encoding for individual mutations in either the NS3 protease domain (3/V132A), or in the NS4A-kink region (4A/L45A, 4A/Y47A, or 4A/L45-Y47-AA) were prepared.
pBici RLuc IRES-Ubi-NS3-3‘ 5B/GAA	Derivative of pBici RLuc IRES-Ubi-NS3-3‘. Inactivated NS5B by mutations GDD to GAA; negative control for RNA replication.
*Trans-complementation assay*	
pCITE NCP7 E2-4A (NS2-3)	Subgenomic expression plasmid encoding for wild-type BVDV-1 NCP7 E2, p7, NS2-3, and 4A (E2-4A). Gene expression is under control of a T7-promotor [69].
pCITE NCP7 E2-4A (NS2-Ubi-NS3)	Derivative of pCITE NCP7 E2-4A (NS2-3). NS2 and NS3 are separated by one monomer of ubiquitin originating from BVDV-strain Osloss. Gene expression is under control of a T7-promotor. Further derivatives of pCITE NCP7 E2-4A (NS2-Ubi-NS3) encoding for individual mutations in NS2 (2/E440V) and in either the NS3 protease domain (3/V132A), or in the NS4A-kink region (4A/L45A, 4A/Y47A, or 4A/L45-Y47-AA) were prepared.

### Vaccinia virus infection, DNA transfection and transient protein expression

The applied procedures have been described [19, 43]. Briefly, Huh7-T7 cells, which express T7 RNA-polymerase [64], or SK6 cells were infected with modified vaccinia virus Ankara-T7pol (MVA-T7pol) [67], subsequently transfected with 4–6 μg of plasmid DNA by using 10 μl Metafectene transfection reagent (Biontex). Protein expression was carried out for 18 h and cells were further processed.

### SDS-PAGE, western blotting and quantitative western blot analyses

Cell lysates were separated with SDS-polyacrylamide-Tricine gels (8 or 10% polyacrylamide) [70]. After electrophoresis, proteins were transferred onto a nitrocellulose membrane. Membranes were blocked with 5% skim milk powder (w/v, Roth) in PBS/Tween 20 (0.05% (vol/vol). Viral proteins were detected with the indicated monoclonal antibodies and visualized with corresponding peroxidase-coupled species-specific secondary antibodies and Western Lightning plus enhanced chemiluminescence reagent (Perkin Elmer, Boston, MA). For the quantification of Western blots, species-specific secondary antibodies coupled to IRdye-800 and an Odyssey SA infrared imaging system were used (*LI-COR* Biosciences, Lincoln, Nebraska, USA).

### *In vitro* transcription and RNA electroporation

2 μg of plasmid DNA were linearized with SmaI (New England Biolabs) and used as template for *in vitro* transcription with MAXIscript SP6 transcription kit (Ambion, Huntingdon, United Kingdom). The amount of RNA was determined by using Quant-iT RNA assay kit and Qubit-Fluorometer (Invitrogen). RNA quality was verified by agarose gel electrophoresis. 1 μg RNA was used for electroporation of 3 x 10^6^ cells. Electroporation of RNAs in MDBK cells was carried out as described previously [71]. After electroporation, cells were transferred into complete MEM and seeded as required for the assay.

### Luciferase assay

Bicistronic reporter constructs, encoding N^pro^-*Renilla* luciferase in the 5´ ORF and one monomer of ubiquitin followed by NS3-5B in the 3´ ORF, were used. RNA replication efficiencies were determined as described in [43]. In short, at each time point (2 h, 24 h, 48 h, 72 h) cells were washed with PBS and lysed in 40 μl lysis-juice (PJK-GmbH). 20 μl of cell lysates were mixed with 100 μl of *Renilla* Glow juice (PJK-GmbH) and measured with a Junior LB 9509 Portable Tube Luminometer (Junior LB9509, Berthold).

### BVDV infection and virus titration

Cell culture supernatants of MDBK-cells were harvested at indicated time points post electroporation (pe) or post infection (pi) and filtered through a 0.2-μm cellulose filter (Sartorius). Infection of MDBK-cells was performed as described previously for 1 h at 37°C [34].

Tissue culture infection dose 50 (TCID_50_/ml) of viral supernatants was determined in three replicates by endpoint titration on MDBK-cells. Viral infection was detected 72 h pi by IF using Mab 8.12.7 directed against NS3/NS2-3. Cy3-labeled anti-mouse secondary antibody was used at 1:1.000. Titration of viral supernatants which were generated during *trans*-complementation assays were detected by IF at 24 h pi.

### Immunofluorescence assay

BVDV-1 infected or electroporated cells were indirectly analyzed for the presence of BVDV-1 NS3/NS2-3 by indirect immunofluorescence assay (IF) with the monoclonal antibody (MAb) 8.12.7 [61]. At indicated time points, cells were washed with 1 x PBS and fixed with 2% paraformaldehyde for 20 min at 4°C. Then, cells were permeabilized by 0.5% n-octyl-β-d-glycopyranoside for 7 min. Cells were washed with 1 x PBS/Tween 20 (0.05% (vol/vol)) and incubated with hybridoma supernatant of anti-NS3 mab at a dilution of 1:40 in PBS/Tween 20 (0.05% (vol/vol)). As secondary antibody, goat anti-mouse IgG conjugated with Cy3 was used at 1:1.000. Images were obtained with a Zeiss Axio Observer.

### *Trans*-complementation assay

Approximately, 5x10^6^ SK6-cells were electroporated with 1 μg of RNA of NCP7 NS2-Ubi-NS3 and seeded into one well of a 6-well plate. 6 h pe cells were infected with MVA-T7pol virus for 1 h at 37°C. Subsequently, cells were transfected with 8 μg of plasmid DNA and incubated for 18 h. Cell culture supernatants were harvested and filtered through a 0.2-μm cellulose filter (Sartorius). In order to test the supernatants for rescued virus, naïve MDBK-cells were inoculated with the filtered cell culture supernatants and incubated for 24 h. NS3-positive cells were detected by anti-NS3 IF. Viral titers were determined as described above in TCID_50_/ml.

### Illustrations

Figs 1B and 2A were prepared using Ribbons and PyMOL (http://pymol.sourceforge.net) softwares, respectively.

### Accession numbers

Atomic coordinates and structure factors amplitudes of NS4A_37_NS3 have been deposited at the Protein Data Bank (PDB) under the accession code 5LKL.

### Statistics

Initially, a Kruskal-Wallis test was performed to compare all groups for differences. In case p < 0.05, pairwise differences between WT and all other mutants were tested using Mann-Whitney tests and interpreted as significant if p < 0.05. For the calculations of the RNA replication assay (S6 Fig), again Kruskal-Wallis tests were performed to test for differences between the groups WT, 3/V132A, 4A/L45A, 4A/Y47A, 4A/L45-Y47-AA and 5B/GAA at each time point separately and regarded to be significant if p < 0.05. In these cases, pairwise differences between WT and all other mutants were again tested using Mann-Whitney tests. Calculations were performed with GraphPad Prism (La Jolla, CA) and IBM SPSS Statistics for Macintosh, Version 22.0 (Armonk, NY: IBM Corp).

## Supporting information

S1 Fig CSFV NS4A_37_NS3 has serine protease activity. (A) Scheme of the constructs used for the *in vivo trans*-cleavage assay. pET 11 NS4A_37_NS3 encodes a single chain version of the NS3-4A protease; pCITE NS5A-5B-myc encodes a BVDV-derived protease substrate. In both constructs the cDNAs are under the control of a T7-RNA polymerase promotor. Individual cleavage products NS5A and NS5B-myc are indicated. (B) NS4A_37_NS3 *trans*-cleavage assay. Vaccinia MVA-T7pol based expression of wild-type NS4A_37_NS3 (WT) and its proteolytically inactive derivative (3/S163A) was performed either without or with co-expression of the NS5A-5B-myc substrate in Huh7-T7 cells. SDS-PAGE and Western Blot analyses of cell lysates were performed. NS4A_37_NS3 expression was detected by anti-NS3 antibody. The NS4A_37_NS3-mediated cleavage of the NS5A-5B-myc substrate was visualized with antibodies directed against NS5A and myc-tag. Molecular mass markers are indicated in kilodaltons (kDa) on the left.
(TIF)

S2 Fig Intact NS4A_37_NS3 (S163A) was recovered from the crystals. SDS-PAGE analysis revealed that the protein present in our crystals is migrating as a single band with a molecular mass corresponding to full-length CSFV NS4A_37_NS3 (S163A). The left lane displays prestained molecular mass markers.
(TIF)

S3 Fig Comparison of the crystal structures of CSFV NS4A_37_NS3, HCV NS4A_14_NS3 and DENV4 NS2B_18_NS3. (Top) Full-length NS3 from *Flaviviridae* HCV (PDB accession code: 1CU1), Dengue virus, serotype 4 (DENV4) (PDB accession codes 2VBC and 2WHX, right and left, respectively) and CSFV (this work) where superposed on the NS3 helicase domain to show the different orientations of the protease domain. Color coding is the same as in Fig 1: NS4A (and NS2B) region is blue, the NS3pro domain is red, NS3hel is yellow and the NS3pro active site residues are shown in green. (Bottom) Orthogonal view of top panel. NABG: Nucleic Acid Binding Groove. The helicase domains are labeled D1, D2 and D3.
(TIF)

S4 Fig Structural comparison of NS3pro orthologs. (A) Structure-based alignment (produced using the Multalign and ESPript, version 2.2, programs) of pestivirus NS3 protease with its hepacivirus and flavivirus counterparts. CSFV (strain Alfort; GenBank accession no. J04358.2), HCV (genotype 1b; GenBank accession no. KJ564295.1), and DENV4 (GenBank accession no. KP774959.1) amino acid sequences of NS3 protease were retrieved from GenBank. The residues forming the catalytic triad are marked by an asterisk. The secondary structure elements of the CSFV, HCV, and DENV NS3pro proteins are displayed above the alignment. Identical or chemically similar residues are indicated at each position with a red background or red font, respectively. (B) Structural comparison of cofactor-bound NS3 protease domain from *Flaviviridae*. Cartoon representation of CSFV NS4A_37_NS3 (this study, PDB accession code 5LKL), HCV NS4A_14_NS3 (PDB accession code 1CU1), DENV4 NS2B_18_NS3 (PDB accession number 2VBC). Blue: NS2B and NS4A cofactors; red: NS3 protease domain; green: catalytic triad (His-Asp-Ser); cyan: hydrophobic residues involved in membrane attachment, in stick representation; N: N terminus; C: C terminus.
(TIF)

S5 Fig Single mutations at the NS3/NS4A-kink region interface allow for efficient polyprotein processing. (A) Schematic depiction of BVDV expression plasmid pT7-DI-388. The construct encodes the nonstructural protein N^pro^ followed by NS3-NS5B. N^pro^ generates the authentic N terminus of NS3. NS3-5B: minimal viral replicase. (B) Analysis of viral polyprotein processing. Vaccinia virus MVA-T7pol-mediated expression of the wild-type (WT) polyprotein or its mutant derivatives was performed. For Western blot analyses primary antibodies directed against NS3, NS4A, NS5A, and NS5B were used. One representative experiment out of three replicates is depicted. Polyprotein processing products are indicated on the right. Mock: vaccinia virus MVA-T7pol infected Huh7-T7 cells; 3/S163A: inactive NS3 protease; WT: wild-type. (C) Co-radioimmunoprecipitation (Co-RIP) of NS3/4A after MVA-T7pol-mediated protein expression. Co-RIP was applied for the detection of NS4A since the NS4A mutants were not detected by our NS4A specific monoclonal antibody. 18 h post transfection cells were incubated for 30 min with medium without Cysteine and Methionine for 30 min followed by the addition of 70 μCi ^35^S Cysteine/Methionine. After 2 h of metabolic labeling, Co-RIP with anti-NS3 antibody was performed. The precipitated proteins were separated by SDS-PAGE and detected by phosphorimaging. Molecular mass markers are indicated on the left (kDa).
(TIF)

S6 Fig Statistical analyses of the relative RNA-replication efficiencies of the replicons depicted in Fig 4 at the indicated time points post electroporation (for details see legend Fig 4). Mean values and standard deviations of four experiments are depicted. First, a Kruskal-Wallis test was performed to test for statistical differences within the tested groups at each time point, separately (24 h: p = 0.0007; 48 h: p = 0.0001; 72 h: p = 0.0064). Subsequently, statistically significant differences of the individual mutant group compared to wild-type was calculated by Mann-Whitney test. It should be noted that differences in Kruskal-Wallis tests at 24h, 48h and 72h remain significant even after adjusting for the testing at four time points. Asterisks indicate for a statistically significant difference with p < 0.05. 5B/GAA: RNA replication-deficient NS5B mutant; WT: wild-type; RLUs: relative light units; RLuc: *Renilla luciferase*.
(TIF)

S7 Fig *Trans*-complementation experiments do not lead to the formation of infectious viruses *via* RNA-recombination events. (A) Top: Schematic depiction of BVDV-1 NCP7 NS2-Ubi-NS3 and the pCITE expression constructs. Viral structural proteins (C, E^rns^, E1, E2), and NS-proteins N^pro^, p7-NS5B are indicated. Bottom: Illustration of the *trans*-complementation assay. SK6-cells were electroporated with 1 μg of *in vitro* transcribed NCP7 NS2-Ubi-NS3 RNA. 6 hours pe, cells were infected with MVA-T7pol vaccinia virus and subsequently transfected with 8 μg of the pCITE expression constructs. After 18 hours, supernatants were harvested and filtered. Subsequently, naïve MDBK cells were inoculated with the respective supernatant. 24 h and 96 h later cells were monitored for infected cells by anti-NS3 IF. UTR: untranslated region; Ubi: ubiquitin; CITE: EMCV-IRES. (B) *Trans*-complementation assay. MDBK cells electroporated with NCP7 NS2-Ubi-NS3 RNA were transfected with the indicated plasmids for MVA-T7pol based protein expression. At 24 h post transfection the supernatants were used to inoculate naïve MDBK cells which were analyzed 24 h and 96 h later by anti NS3 IF. One representative assay out of three performed is shown. NS2-3: uncleaved NS2-3 protein.
(TIF)

S1 Table Disordered regions in NS4A_37_NS3 crystal form. (DOCX)
