# Supplementary figures and images for: A positive-strand RNA virus uses alternative protein-protein interactions within a viral protease/cofactor complex to switch between RNA replication and virion morphogenesis
Source: PLoS Pathog. 2017 Feb 2;13(2):e1006134. doi: 10.1371/journal.ppat.1006134 (PMC5308820; doi:10.1371/journal.ppat.1006134)

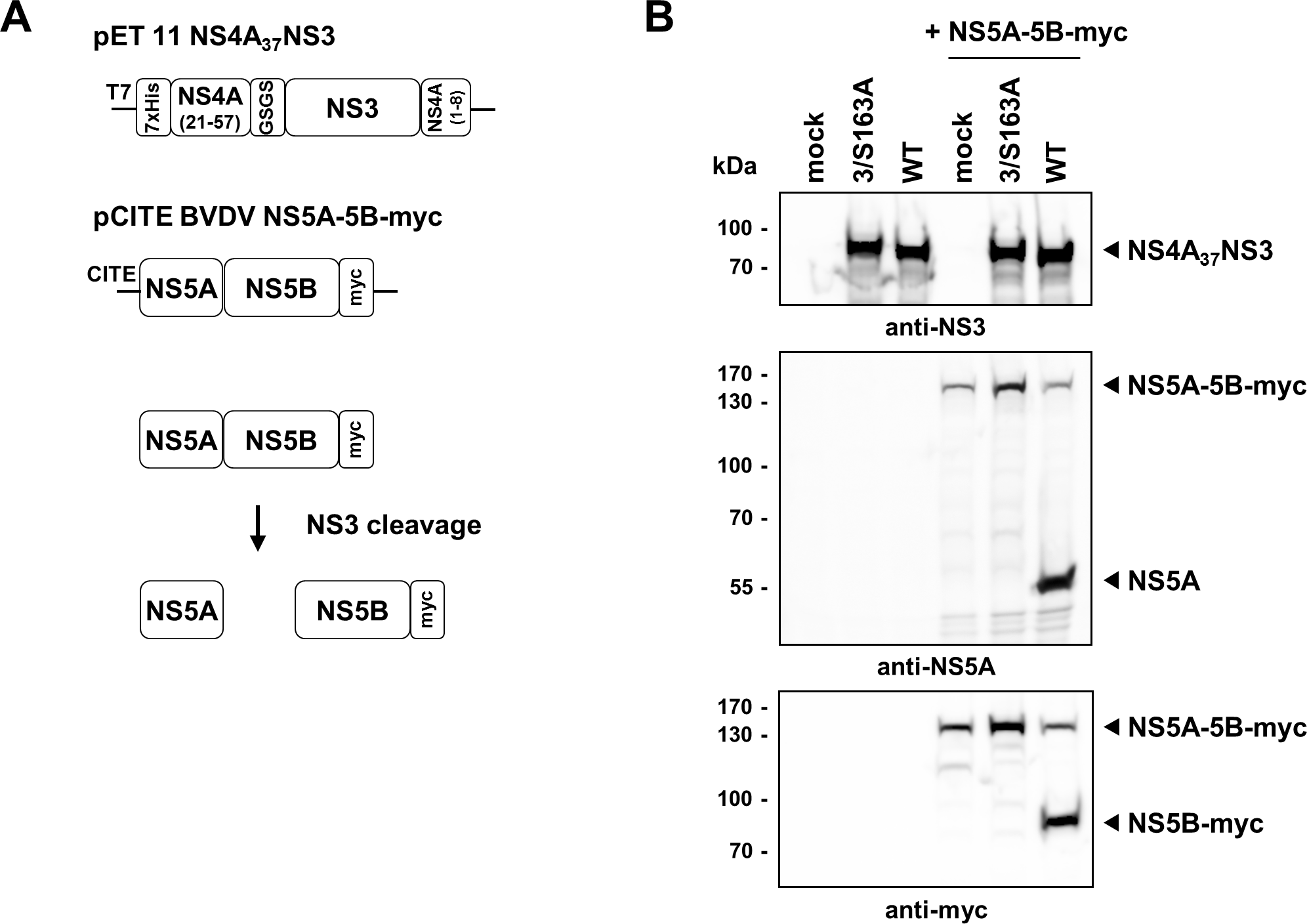

Supplement: S1 Fig — (A) Scheme of the constructs used for the in vivo trans-cleavage assay. pET 11 NS4A37NS3 encodes a single chain version of the NS3-4A protease; pCITE NS5A-5B-myc encodes a BVDV-derived protease substrate. In both constructs the cDNAs are under the control of a T7-RNA polymerase promotor. Individual cleavage products NS5A and NS5B-myc are indicated. (B) NS4A37NS3 trans-cleavage assay. Vaccinia MVA-T7pol based expression of wild-type NS4A37NS3 (WT) and its proteolytically inactive derivative (3/S163A) was performed either without or with co-expression of the NS5A-5B-myc substrate in Huh7-T7 cells. SDS-PAGE and Western Blot analyses of cell lysates were performed. NS4A37NS3 expression was detected by anti-NS3 antibody. The NS4A37NS3-mediated cleavage of the NS5A-5B-myc substrate was visualized with antibodies directed against NS5A and myc-tag. Molecular mass markers are indicated in kilodaltons (kDa) on the left. (TIF) [file ppat.1006134.s001.tif]

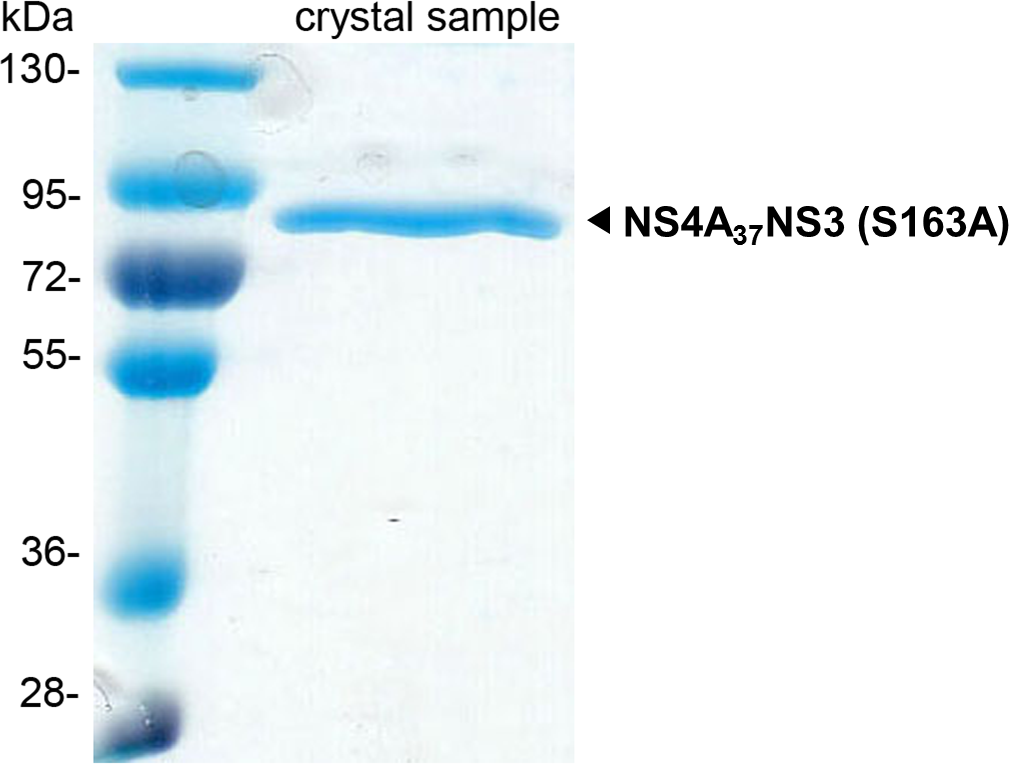

Supplement: S2 Fig — SDS-PAGE analysis revealed that the protein present in our crystals is migrating as a single band with a molecular mass corresponding to full-length CSFV NS4A37NS3 (S163A). The left lane displays prestained molecular mass markers. (TIF) [file ppat.1006134.s002.tif]

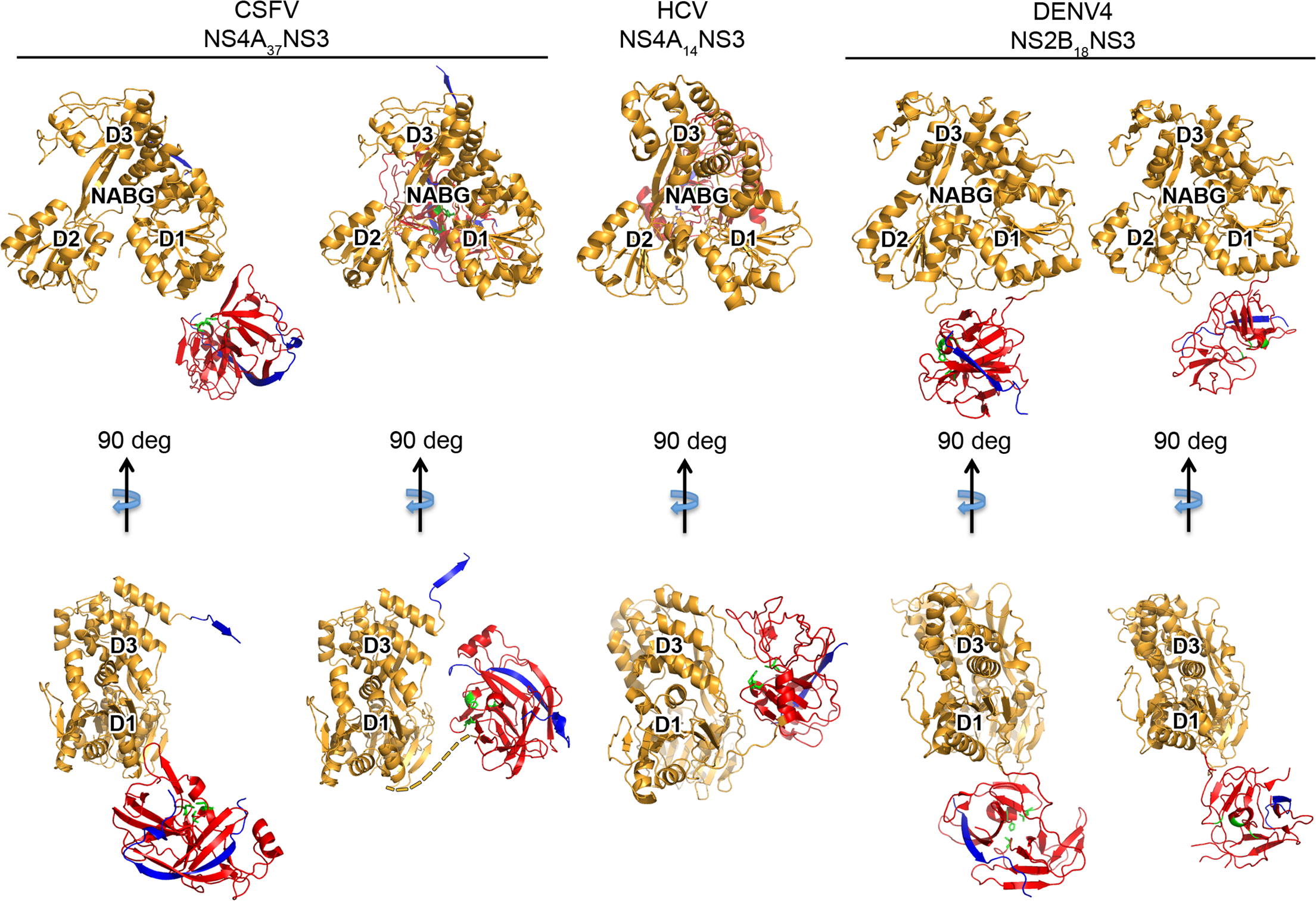

Supplement: S3 Fig — (Top) Full-length NS3 from Flaviviridae HCV (PDB accession code: 1CU1), Dengue virus, serotype 4 (DENV4) (PDB accession codes 2VBC and 2WHX, right and left, respectively) and CSFV (this work) where superposed on the NS3 helicase domain to show the different orientations of the protease domain. Color coding is the same as in Fig 1: NS4A (and NS2B) region is blue, the NS3pro domain is red, NS3hel is yellow and the NS3pro active site residues are shown in green. (Bottom) Orthogonal view of top panel. NABG: Nucleic Acid Binding Groove. The helicase domains are labeled D1, D2 and D3. (TIF) [file ppat.1006134.s003.tif]

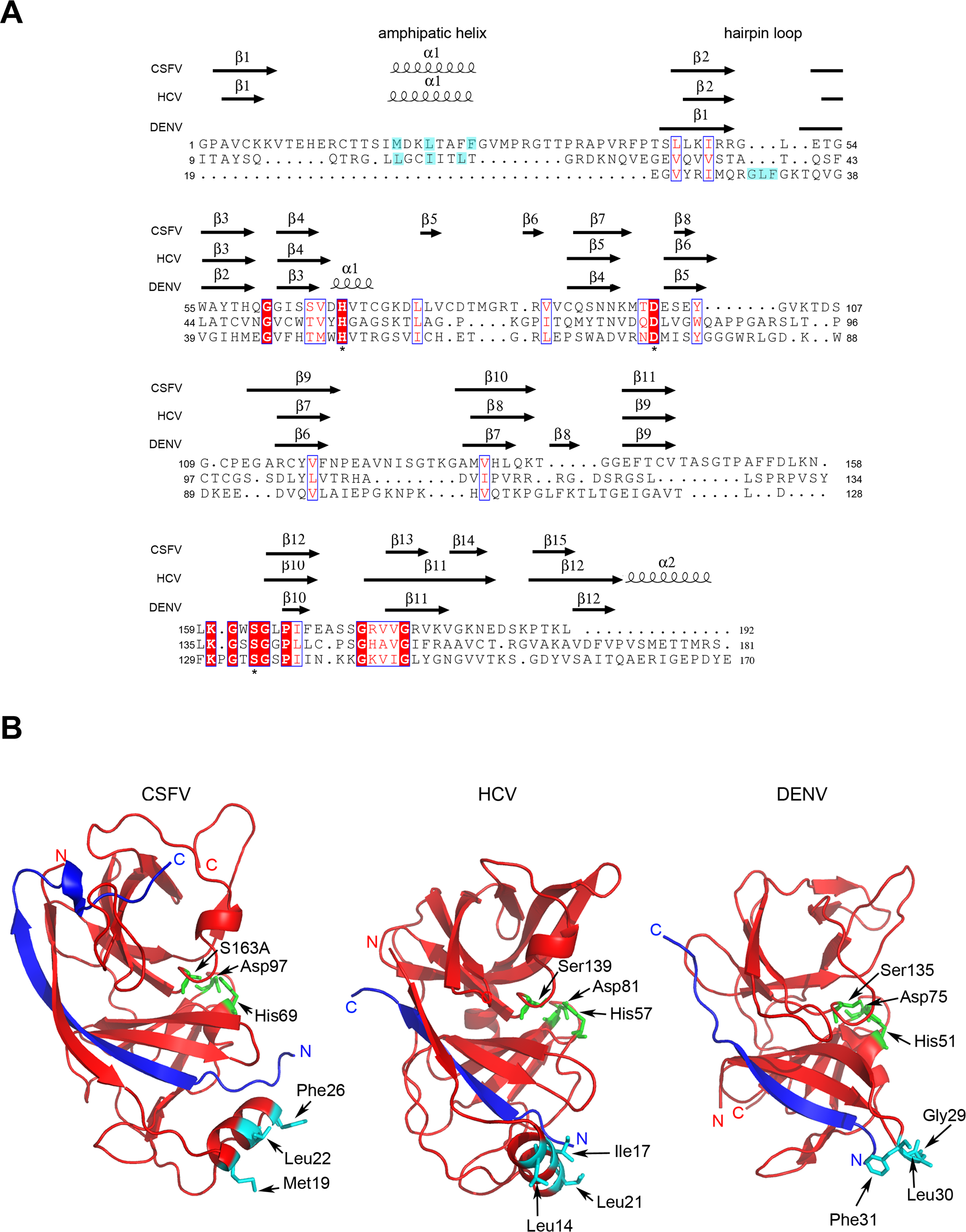

Supplement: S4 Fig — (A) Structure-based alignment (produced using the Multalign and ESPript, version 2.2, programs) of pestivirus NS3 protease with its hepacivirus and flavivirus counterparts. CSFV (strain Alfort; GenBank accession no. J04358.2), HCV (genotype 1b; GenBank accession no. KJ564295.1), and DENV4 (GenBank accession no. KP774959.1) amino acid sequences of NS3 protease were retrieved from GenBank. The residues forming the catalytic triad are marked by an asterisk. The secondary structure elements of the CSFV, HCV, and DENV NS3pro proteins are displayed above the alignment. Identical or chemically similar residues are indicated at each position with a red background or red font, respectively. (B) Structural comparison of cofactor-bound NS3 protease domain from Flaviviridae. Cartoon representation of CSFV NS4A37NS3 (this study, PDB accession code 5LKL), HCV NS4A14NS3 (PDB accession code 1CU1), DENV4 NS2B18NS3 (PDB accession number 2VBC). Blue: NS2B and NS4A cofactors; red: NS3 protease domain; green: catalytic triad (His-Asp-Ser); cyan: hydrophobic residues involved in membrane attachment, in stick representation; N: N terminus; C: C terminus. (TIF) [file ppat.1006134.s004.tif]

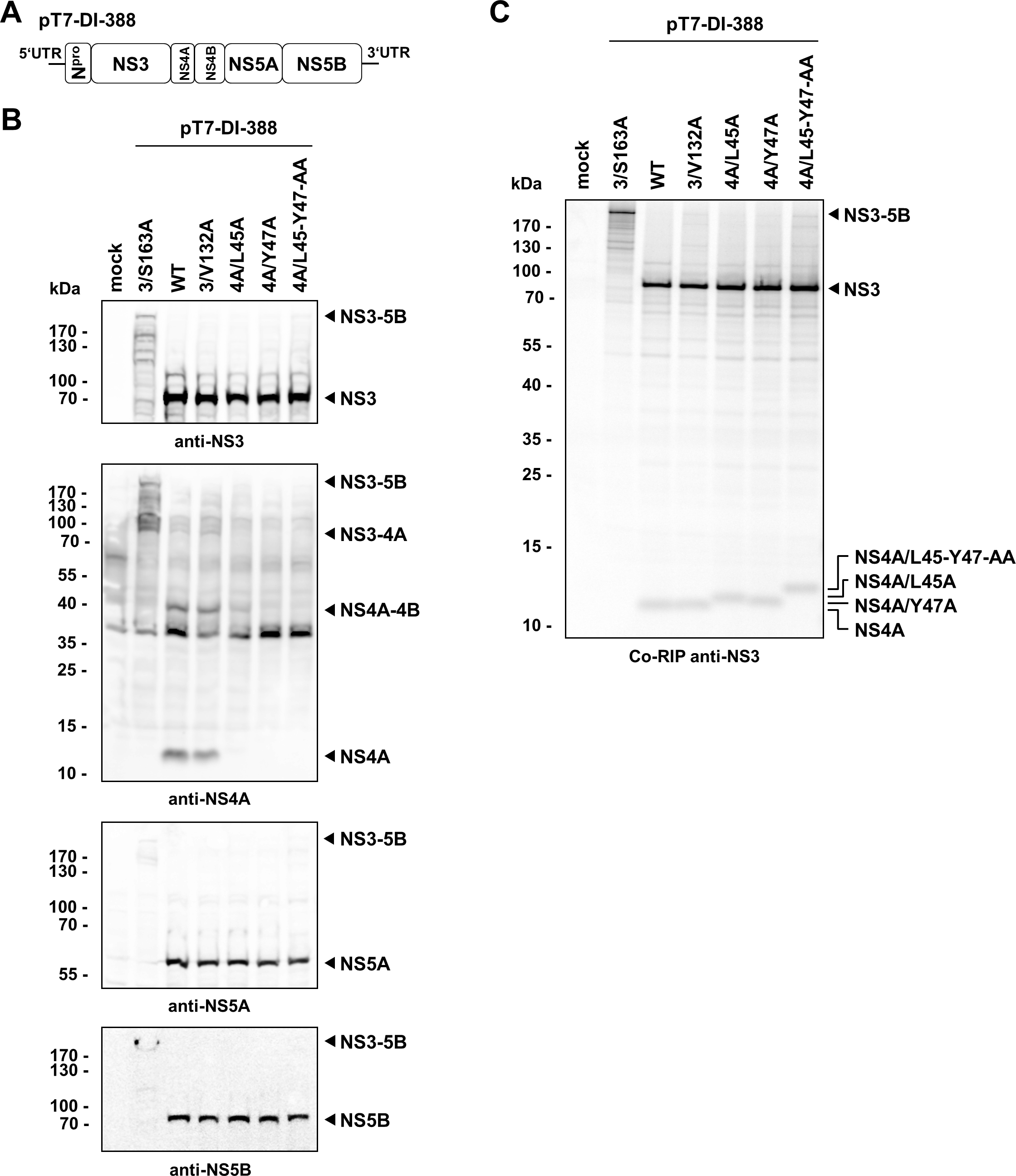

Supplement: S5 Fig — (A) Schematic depiction of BVDV expression plasmid pT7-DI-388. The construct encodes the nonstructural protein Npro followed by NS3-NS5B. Npro generates the authentic N terminus of NS3. NS3-5B: minimal viral replicase. (B) Analysis of viral polyprotein processing. Vaccinia virus MVA-T7pol-mediated expression of the wild-type (WT) polyprotein or its mutant derivatives was performed. For Western blot analyses primary antibodies directed against NS3, NS4A, NS5A, and NS5B were used. One representative experiment out of three replicates is depicted. Polyprotein processing products are indicated on the right. Mock: vaccinia virus MVA-T7pol infected Huh7-T7 cells; 3/S163A: inactive NS3 protease; WT: wild-type. (C) Co-radioimmunoprecipitation (Co-RIP) of NS3/4A after MVA-T7pol-mediated protein expression. Co-RIP was applied for the detection of NS4A since the NS4A mutants were not detected by our NS4A specific monoclonal antibody. 18 h post transfection cells were incubated for 30 min with medium without Cysteine and Methionine for 30 min followed by the addition of 70 μCi 35S Cysteine/Methionine. After 2 h of metabolic labeling, Co-RIP with anti-NS3 antibody was performed. The precipitated proteins were separated by SDS-PAGE and detected by phosphorimaging. Molecular mass markers are indicated on the left (kDa). (TIF) [file ppat.1006134.s005.tif]

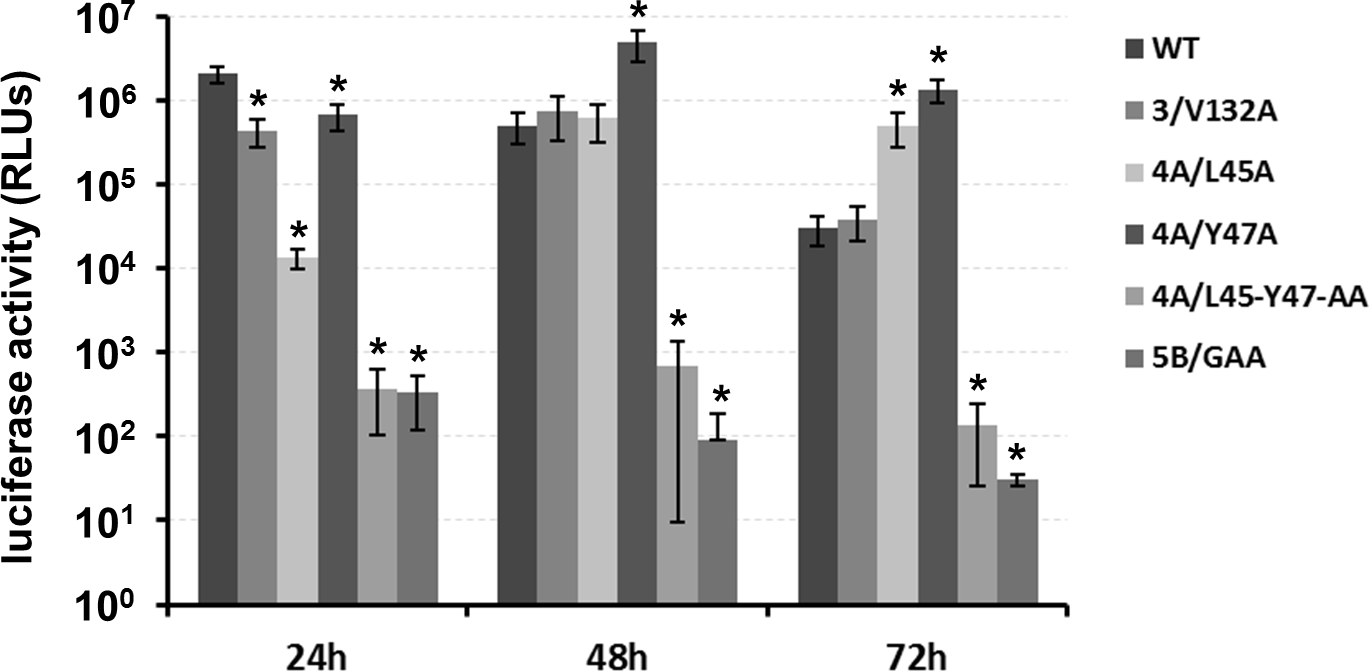

Supplement: S6 Fig — Mean values and standard deviations of four experiments are depicted. First, a Kruskal-Wallis test was performed to test for statistical differences within the tested groups at each time point, separately (24 h: p = 0.0007; 48 h: p = 0.0001; 72 h: p = 0.0064). Subsequently, statistically significant differences of the individual mutant group compared to wild-type was calculated by Mann-Whitney test. It should be noted that differences in Kruskal-Wallis tests at 24h, 48h and 72h remain significant even after adjusting for the testing at four time points. Asterisks indicate for a statistically significant difference with p < 0.05. 5B/GAA: RNA replication-deficient NS5B mutant; WT: wild-type; RLUs: relative light units; RLuc: Renilla luciferase. (TIF) [file ppat.1006134.s006.tif]

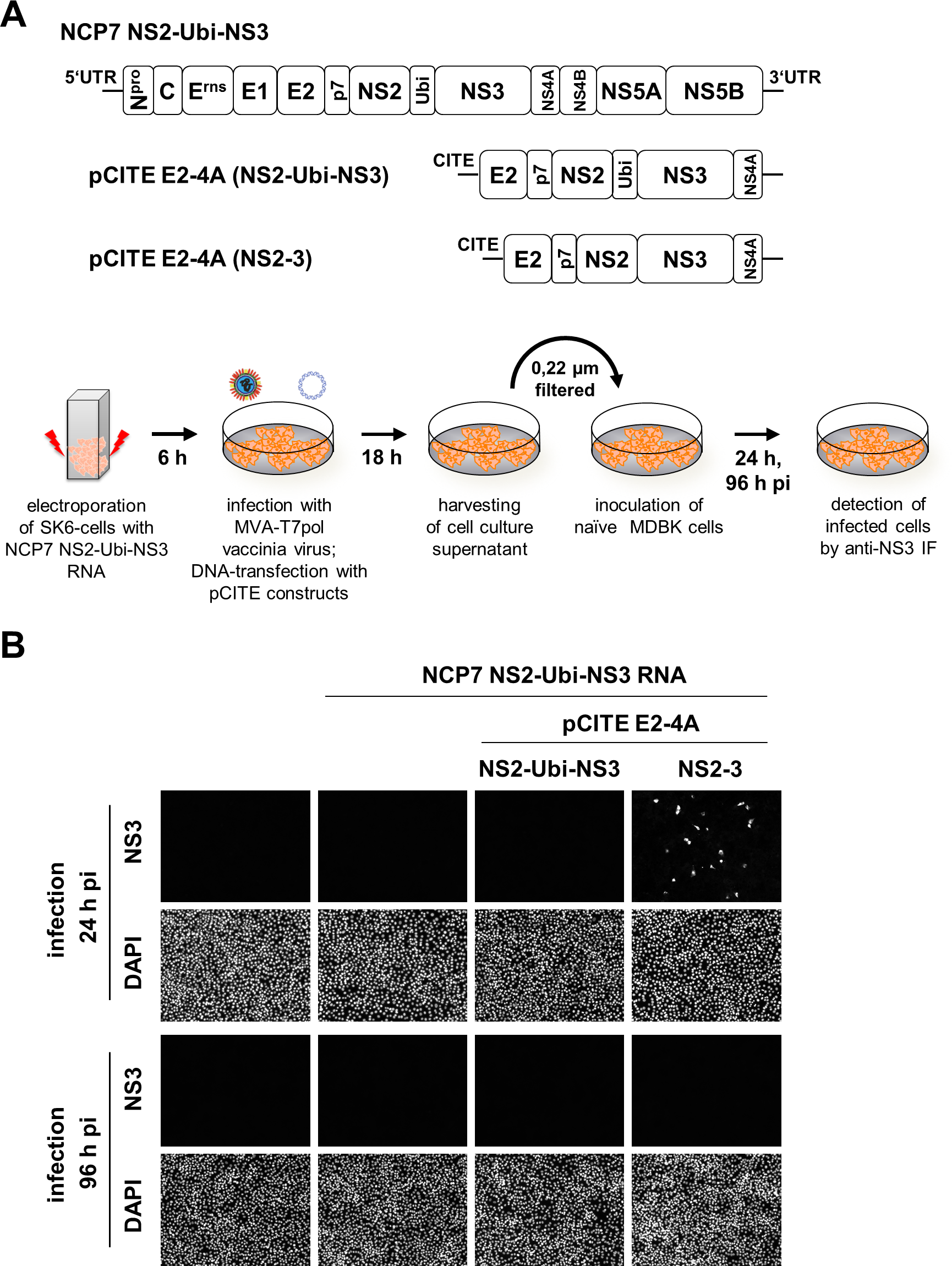

Supplement: S7 Fig — (A) Top: Schematic depiction of BVDV-1 NCP7 NS2-Ubi-NS3 and the pCITE expression constructs. Viral structural proteins (C, Erns, E1, E2), and NS-proteins Npro, p7-NS5B are indicated. Bottom: Illustration of the trans-complementation assay. SK6-cells were electroporated with 1 μg of in vitro transcribed NCP7 NS2-Ubi-NS3 RNA. 6 hours pe, cells were infected with MVA-T7pol vaccinia virus and subsequently transfected with 8 μg of the pCITE expression constructs. After 18 hours, supernatants were harvested and filtered. Subsequently, naïve MDBK cells were inoculated with the respective supernatant. 24 h and 96 h later cells were monitored for infected cells by anti-NS3 IF. UTR: untranslated region; Ubi: ubiquitin; CITE: EMCV-IRES. (B) Trans-complementation assay. MDBK cells electroporated with NCP7 NS2-Ubi-NS3 RNA were transfected with the indicated plasmids for MVA-T7pol based protein expression. At 24 h post transfection the supernatants were used to inoculate naïve MDBK cells which were analyzed 24 h and 96 h later by anti NS3 IF. One representative assay out of three performed is shown. NS2-3: uncleaved NS2-3 protein. (TIF) [file ppat.1006134.s007.tif]
