# Supplementary material for: A positive-strand RNA virus uses alternative protein-protein interactions within a viral protease/cofactor complex to switch between RNA replication and virion morphogenesis
Source: PLoS Pathog. 2017 Feb 2;13(2):e1006134. doi: 10.1371/journal.ppat.1006134 (PMC5308820; doi:10.1371/journal.ppat.1006134)
Supplement: S1 Table — (DOCX) [file ppat.1006134.s008.docx]

**S1 Table Disordered regions in NS4A_37_NS3 crystal form.**

| Chain* | A | B |
| --- | --- | --- |
| Disordered region |  |  |
| NS3 Helicase domain  N-terminal  Linker D1-D2  Domain 2 | 4-9  166-170  182-189 | 6-12  159-172  185-187  248-255  263-272  315-321 |
| NS4A cofactor | 50-57 | 50-57 |

*In both chains, A and B, of CSFV NS4A_37_NS3 NS3h crystal form, the N-terminal histidine tag and the TEV protease cleavage site are disordered. The reported structures contain all amino acids except those listed here.
